# Functional roles of pantothenic acid, riboflavin, thiamine, and choline in adipocyte browning in chemically induced human brown adipocytes

**DOI:** 10.1038/s41598-024-69364-w

**Published:** 2024-08-06

**Authors:** Yukimasa Takeda, Ping Dai

**Affiliations:** Department of Cellular Regenerative Medicine, Graduate School of Medical Science, Kyoto Prefectural University of Medicine, 465 Kajii-Cho, Kawaramachi-Hirokoji, Kamigyo-Ku, Kyoto, 602-8566 Japan

**Keywords:** Brown adipocytes, Vitamin B, Choline, Pantothenic acid, Riboflavin, Thiamine, Cell biology, Molecular biology

## Abstract

Brown fat is a therapeutic target for the treatment of obesity-associated metabolic diseases. However, nutritional intervention strategies for increasing the mass and activity of human brown adipocytes have not yet been established. To identify vitamins required for brown adipogenesis and adipocyte browning, chemical compound-induced brown adipocytes (ciBAs) were converted from human dermal fibroblasts under serum-free and vitamin-free conditions. Choline was found to be essential for adipogenesis. Additional treatment with pantothenic acid (PA) provided choline-induced immature adipocytes with browning properties and metabolic maturation, including uncoupling protein 1 (UCP1) expression, lipolysis, and mitochondrial respiration. However, treatment with high PA concentrations attenuated these effects along with decreased glycolysis. Transcriptome analysis showed that a low PA concentration activated metabolic genes, including the futile creatine cycle-related thermogenic genes, which was reversed by a high PA concentration. Riboflavin treatment suppressed thermogenic gene expression and increased lipolysis, implying a metabolic pathway different from that of PA. Thiamine treatment slightly activated thermogenic genes along with decreased glycolysis. In summary, our results suggest that specific B vitamins and choline are uniquely involved in the regulation of adipocyte browning via cellular energy metabolism in a concentration-dependent manner.

## Introduction

Obesity is associated with an increased risk of adverse health outcomes^1^. Mammals possess brown adipose tissue (BAT) as a thermogenic organ to maintain body temperature and metabolic homeostasis^2^. BAT specialises in the consumption of fatty acids and glucose to convert chemical energy into heat, in a process called non-shivering thermogenesis^3^. Thermogenesis in BAT contributes to the maintenance of body temperature in cold environments, which is uniquely conferred by uncoupling protein 1 (UCP1) located on the inner mitochondrial membrane. UCP1 dissipates the mitochondrial H^+^ gradient by conducting H^+^ across the inner membrane to produce heat^4^. In adult humans, metabolically active BAT exists in the cervical and supraclavicular areas^5,6^. Its prevalence is inversely correlated with body mass index (BMI) and aging, implying that the disappearance of BAT may be associated with the development of obesity and related metabolic diseases^7^. Therefore, human BAT is an attractive therapeutic target for the prevention and treatment of metabolic diseases^8^. Despite the importance of its thermogenic and metabolic functions, a sustainable and side effect-free strategy to enhance the amount of brown adipocytes has not been sufficiently developed^9^. Activation of human brown adipocytes by ingesting specific micronutrients is a promising long-term strategy for the management of obesity and metabolic disorders^10^.

Vitamins play a pivotal role in cellular energy metabolism and the prevention of metabolic diseases^11^. Thiamine, riboflavin, and pantothenic acid (PA), categorised as B vitamins, serve as cofactors for hundreds of enzymes that are critical for cellular and mitochondrial energy metabolism^12,13^. Coenzyme A (CoA) synthesised from PA is required for anabolic reactions, such as the synthesis of fatty acids, cholesterols, acetylcholine, and bile acids. CoA is also involved in catabolic reactions for the efficient production of adenosine triphosphate (ATP) in the mitochondria using substrates such as fatty acids, carbohydrates, and amino acids. In particular, CoA is an essential cofactor of pyruvate dehydrogenase (PDH) which connects glycolysis and the carboxylic acid (TCA) cycle by converting pyruvate to acetyl-CoA. PA is a dietary supplement used to treat nutrient deficiencies; however, its effects on obesity and metabolic diseases are controversial. Several lines of evidence have suggested that high B vitamin intake from vitamin-fortified foods and drinks promoted body fat gain and was correlated with the prevalence of obesity and diabetes^14–16^. An epidemiological report on children and adolescents indicated that the intake of four vitamins (thiamine, pyridoxine, niacin, and pantothenic acid) increased the likelihood of obesity, whereas riboflavin had no significant association^17^. In contrast, a recent study indicated that orally administered PA protected mice against high-fat diet-induced weight gain and reduced subcutaneous and hepatic fats^18^. Another study on the association between micronutrients and visceral fat accumulation indicated that PA ingestion was negatively correlated with visceral fat area in healthy Japanese adults^19^. Furthermore, the administration of pantethine, an intermediate in the production of CoA, lowered cardiovascular disease risk markers such as low-density lipoprotein cholesterol and total cholesterol without a significant change in BMI during the study^20^. Thus, PA intake is diversely involved in the pathogenesis of obesity and metabolic diseases.

Due to the limited availability of primary brown fat, a method for converting primary human dermal fibroblasts (HDFs) into brown adipocytes was developed^21–23^. A chemical cocktail (RoFB) consisting of rosiglitazone, forskolin, and bone morphogenetic protein 7 (BMP7) was continuously supplied under a serum-free condition in the conversion of HDFs to chemical compound-induced brown adipocytes (ciBAs). In our previous study, ciBAs more abundantly expressed UCP1 than adipocytes differentiated from mesenchymal stem cells (MSCs)^24^. The effects of capsaicin, carnitine, and free fatty acids on thermogenic functions and mitochondrial energy metabolism in ciBAs under serum-free conditions have also been reported^25,26^. These studies suggest that ciBAs serve as a human brown adipocyte model that is useful for uncovering the effects of bioactive molecules on adipocyte browning^10^. Due to the essential roles of vitamins in organisms, it is difficult to evaluate their functions under specific vitamin-free conditions in vivo. Furthermore, in vitro cell culture systems generally use a basal medium and serum containing numerous nutrients and vitamins. For example, Dulbecco's Modified Eagle Medium (DMEM) generally includes eight soluble vitamins and vitamin-like compounds, such as choline chloride (4.0 μg/mL), D-calcium pantothenate (4.0 μg/mL), folic acid (4.0 μg/mL), nicotinamide (4.0 μg/mL), pyridoxine hydrochloride (4.0 μg/mL), riboflavin (0.4 μg/mL), thiamine hydrochloride (4.0 μg/mL), and myo-inositol (7.2 μg/mL). In this study, the serum-free method using a custom medium without these vitamins allowed for specific vitamin-free conditions for the conversion of HDFs into ciBAs. We aimed to evaluate the precise effects of these soluble vitamins on brown adipocytes to gain further insights into nutritional interventions to increase the mass and activity of brown adipocytes in the body.

## Results

### Choline and pantothenic acid are uniquely required for adipogenesis and adipocyte browning, respectively

**Figure 1:**
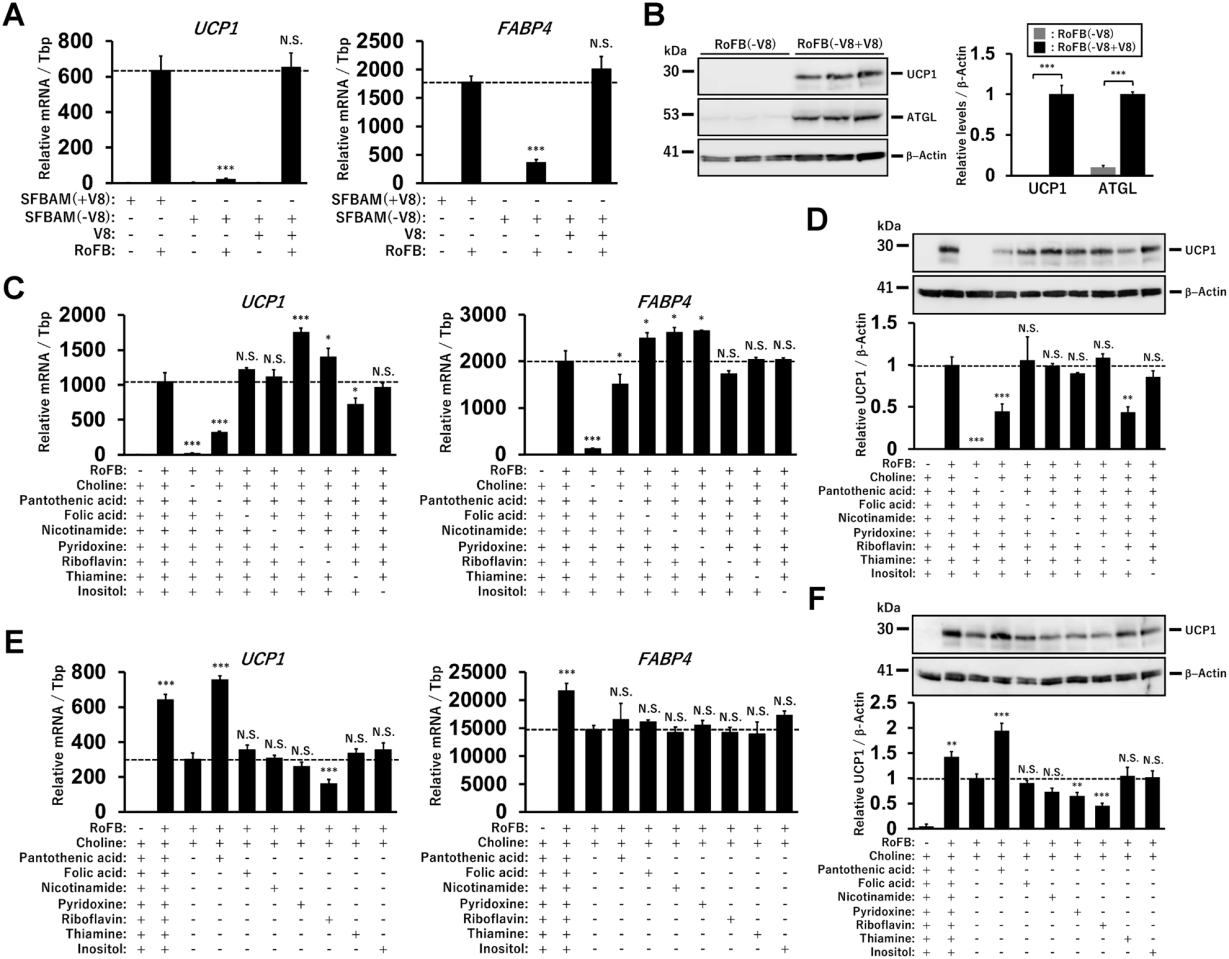
Identification of vitamins required for the conversion of HDFs to ciBAs. (**A**) The expression of *UCP1* and *FABP4* was measured by qRT-PCR analysis in ciBAs converted using the serum-free brown adipogenic medium (SFBAM) in the presence or absence of eight vitamins (V8) included in the basal culture medium throughout the conversion. One-way ANOVA with Tukey’s multiple comparison tests was performed by comparing each value with the one under the condition of SFBAM(+ V8) including RoFB, as indicated by dashed bars in the figures. (**B**) The protein levels of UCP1, ATGL, and β-Actin were quantified by immunoblotting in the ciBAs. The band intensities were quantified by densitometry using ImageJ software. β-Actin was used as a loading control for normalization. (**C**) The expression of *UCP1* and *FABP4* was measured in ciBAs converted by SFBAM excluding each of the eight vitamins. One-way ANOVA with Tukey’s multiple comparison tests was performed by comparing each value with the one under the condition of RoFB and all the vitamins, as indicated by dashed bars in the figures. (**D**) The protein levels of UCP1 and β-Actin were quantified by immunoblotting in the ciBAs. (**E**) The expression of *UCP1* and *FABP4* was measured in ciBAs converted by SFBAM only containing choline and another vitamin. One-way ANOVA with Tukey’s multiple comparison tests was performed by comparing each value with the one under the condition of RoFB and only choline, as indicated by dashed bars in the figures. (**F**) The protein levels of UCP1 and β-Actin were quantified by immunoblotting in the ciBAs. Data represent mean ± SD (*n* = 3). One-way ANOVA with Tukey’s multiple comparison tests: * *p* < 0.05, ** *p* < 0.01, *** *p* < 0.001, N.S.; not significant.

To assess the requirement of each vitamin for brown adipogenesis and adipocyte browning, ciBAs were converted from HDFs using serum-free brown adipogenic medium (SFBAM) without all eight vitamins (V8) included in DMEM^23^. Immunocytochemical analysis showed that primary HDFs were successfully converted into ciBA with fluorescent signals for lipid droplets and UCP1 protein through treatment with the chemical cocktail, RoFB (Supplementary Fig. 1A). The expression of *UCP1*, a brown adipocyte-specific gene, and *FABP4*, an adipocyte-enriched gene, was induced by RoFB, but not in cells cultured without RoFB (Fig. 1A). However, the expression was robustly reduced in HDFs treated with RoFB in the vitamin-free medium, SFBAM(-V8), suggesting that the conversion into ciBAs was largely abolished under vitamin-free conditions. The addition of the eight vitamins (V8) to SFBAM(-V8) recovered the expression of *UCP1* and *FABP4*. Immunoblotting analysis showed that the protein expression of UCP1 and ATGL, an adipocyte-enriched protein, was induced by RoFB in the presence of the eight vitamins (Fig. 1B). Loss of each one of the eight vitamins indicated that choline (Ch) was required for adipogenesis during the conversion because *FABP4* expression was strongly repressed (Fig. 1C). Furthermore, the absence of either pantothenic acid (PA) or thiamine reduced *UCP1* expression, suggesting that they may be required for adipocyte browning. UCP1 protein expression was similarly reduced by each of them, corresponding to the mRNA levels (Fig. 1D). Each vitamin was then added to the medium containing only Ch, which was minimally required for adipogenesis (Fig. 1E). *UCP1* expression was uniquely elevated by additional treatment with PA without a change in *FABP4* expression. In contrast, the addition of riboflavin reduced *UCP1* expression. Immunoblotting analysis confirmed that UCP1 protein increased and decreased in response to the additional treatment with PA and riboflavin, respectively (Fig. 1F).

**Figure 2:**
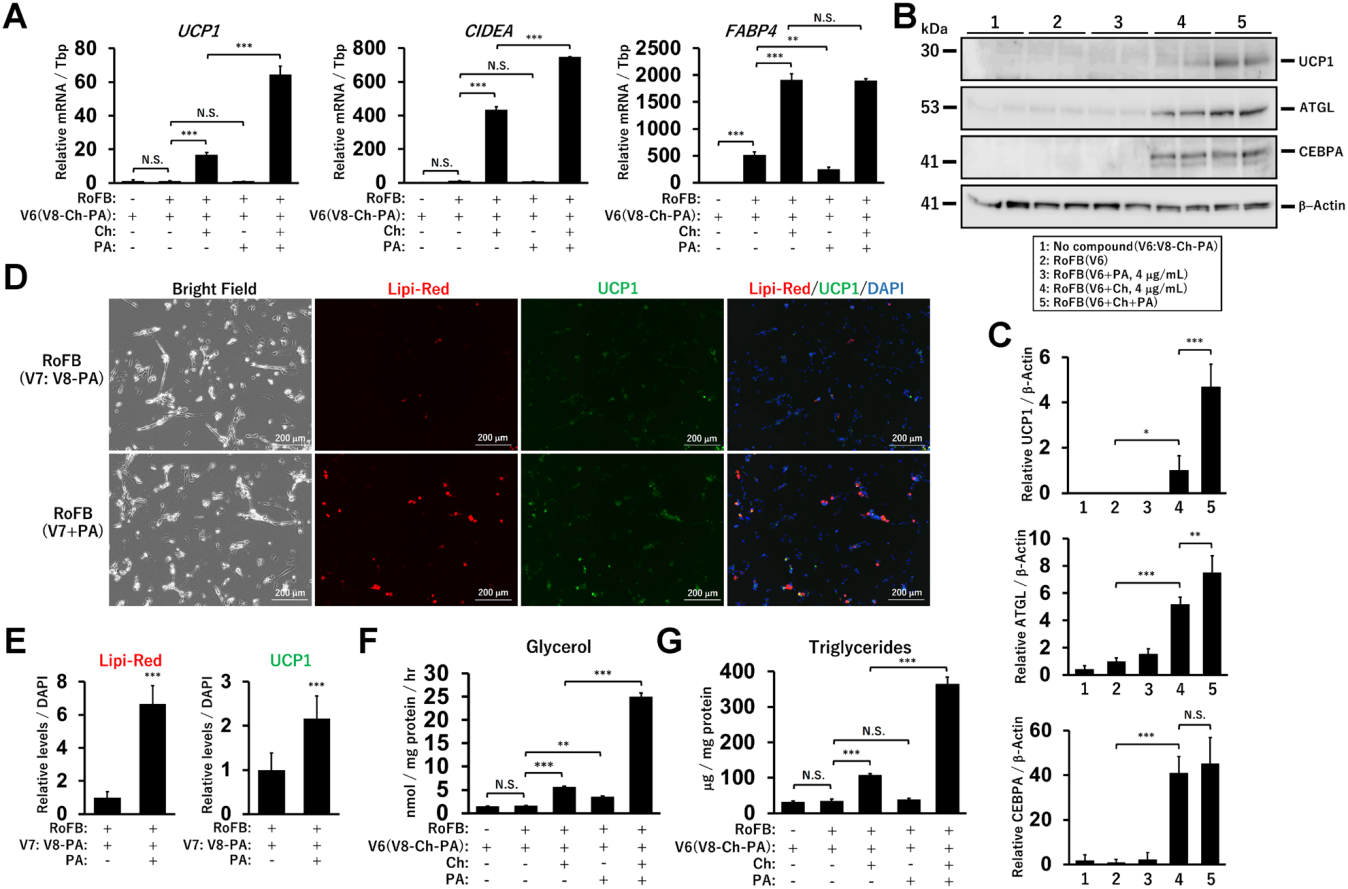
Effects of choline and pantothenic acid on UCP1 expression and lipid metabolism in ciBAs. (**A**) The expression of *UCP1*, *CIDEA*, and *FABP4* was measured by qRT-PCR analysis in ciBAs converted by the serum-free medium in the presence or absence of choline (Ch) and pantothenic acid (PA) throughout the conversion, as indicated. (**B**) The protein levels of UCP1, ATGL, CEBPA, and β-Actin were quantified by immunoblotting in the ciBAs. (**C**) The band intensities were quantified by densitometry using ImageJ software. β-Actin was used as a loading control for normalization. (**D**) Representative images of bright field, lipid droplets stained by Lipi-Red (red), UCP1 expression (green), and merged image in the ciBAs converted in the presence or absence of PA throughout the conversion. The nuclei were visualised by DAPI (blue). Scale bars represent 200 μm. (**E**) The area of the staining for lipid droplets and UCP1 was quantified by ImageJ software. *P* values were determined using student’s t-test. (**F,G**) Glycerol secretion and triglyceride accumulation were measured in ciBAs converted under each condition. Data represent mean ± SD (*n* = 3). One-way ANOVA with Tukey’s multiple comparison tests: * *p* < 0.05, ** *p* < 0.01, *** *p* < 0.001, N.S.; not significant.

In the presence of the other six vitamins, Ch and PA were required for the conversion to ciBAs (Fig. 2A). The combination of Ch and PA synergistically activated the expression of *UCP1* and *CIDEA*, another brown adipocyte-enriched gene. The treatment with PA only did not activate *FABP4* expression, consistent with the observation that Ch, but not PA, was required for adipogenesis. The removal of Ch and/or PA for two days before harvest partially reduced *UCP1* and *FABP4* expression, indicating that these deficiencies might affect the transcription within a few days (Supplementary Fig. S1B,C). Immunoblotting showed that UCP1, ATGL, and CEBPA protein levels were increased by Ch treatment (Fig. 2B,C). Consistent with *UCP1* mRNA levels, the combination of Ch and PA synergistically activated UCP1 expression. Immunocytochemical analysis showed that PA treatment enhanced the staining of lipid droplets and UCP1 (Fig. 2D,E). Glycerol secretion and triglyceride accumulation were enhanced by Ch treatment, likely owing to adipocyte generation (Fig. 2F,G). In addition, they were both enhanced by the additional treatment with PA, suggesting that PA treatment provided Ch-induced immature ciBAs with robust UCP1 expression and metabolic maturation, such as lipolysis and triglyceride storage.

### Pantothenic acid controls UCP1 expression and lipid metabolism in a concentration-dependent manner

**Figure 3:**
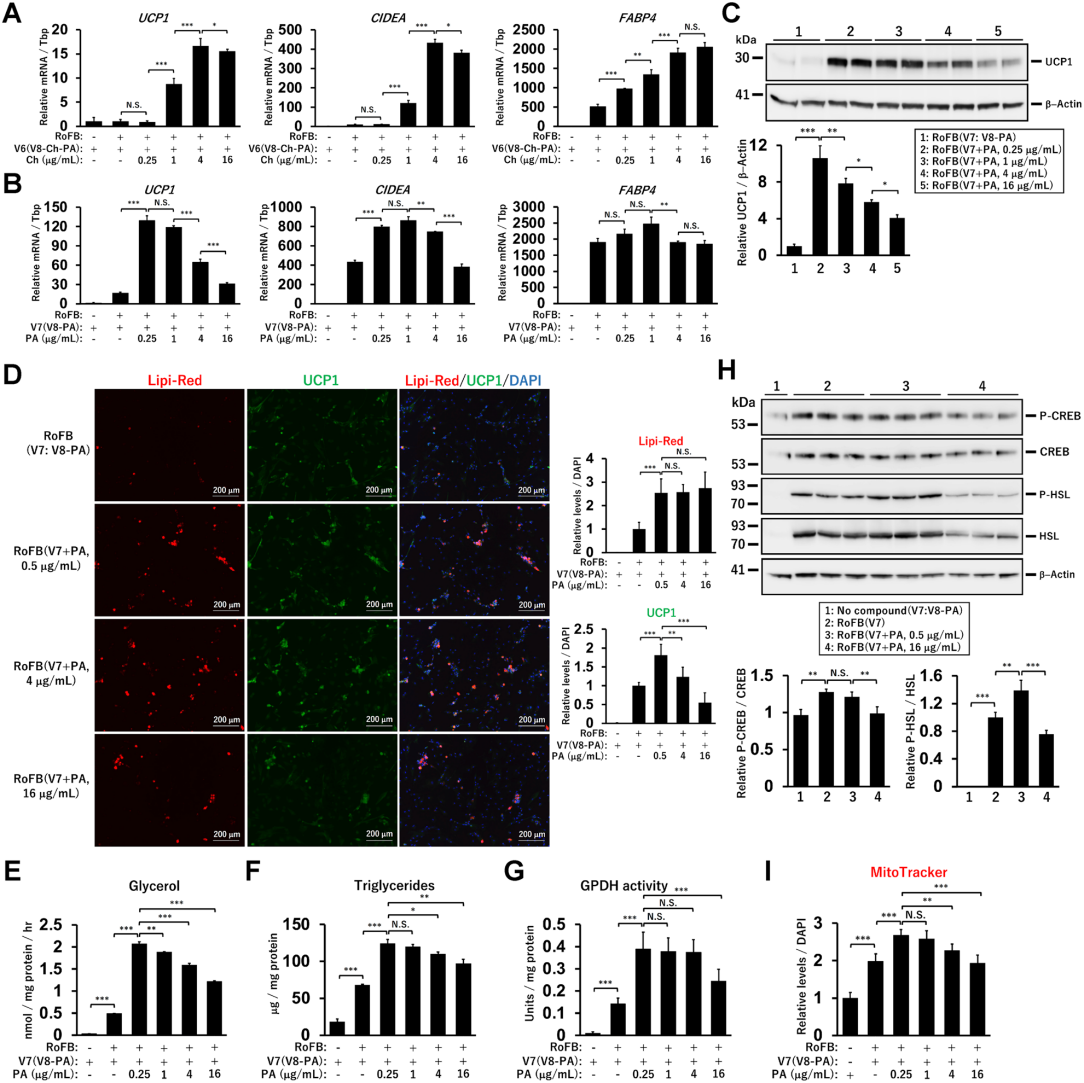
Concentration-dependent effects of PA on *UCP1* expression in ciBAs. (**A**) The expression of *UCP1*, *CIDEA*, and *FABP4* was measured by qRT-PCR analysis in ciBAs treated with Ch at concentrations from 0.25 to 16 μg/mL throughout the experiments. (**B**) The expression was measured in ciBAs treated with PA at concentrations from 0.25 to 16 μg/mL. (**C**) UCP1 and β-Actin proteins were detected by immunoblotting analysis in ciBAs treated with PA at various concentrations. (**D**) Representative images of Lipi-Red staining (red), UCP1 expression (green), and DAPI (blue) in the ciBAs treated with PA at various concentrations throughout the experiments. The area of the staining for Lipi-Red and UCP1 was quantified by ImageJ software. (**E**–**G**) Glycerol secretion, triglyceride accumulation, and glycerol-3-phosphate dehydrogenase 1 (GPDH) activity were measured in ciBAs treated with PA at various concentrations. (**H**) The phosphorylation of CREB and HSL proteins was quantified by immunoblotting analysis in ciBAs treated with PA at 0.5 μg/mL and 16 μg/mL throughout the experiments. The band intensities were quantified by densitometry using ImageJ software. (**I**) Cellular mitochondria contents were evaluated by MitoTracker staining in ciBAs treated with PA at various concentrations. Data represent mean ± SD (*n* = 3). One-way ANOVA with Tukey’s multiple comparison tests: * *p* < 0.05, ** *p* < 0.01, *** *p* < 0.001, N.S.; not significant.

Subsequently, the effects of various Ch and PA concentrations were assessed. The expression of *UCP1*, *CIDEA*, and *FABP4* was increased in a dose-dependent manner from 0.25 to 4 μg/mL and almost reached a plateau (Fig. 3A). In contrast, low PA concentrations (0.25 and 1 μg/mL) robustly induced the expression of *UCP1* and *CIDEA*, while higher concentrations (4 and 16 μg/mL) repressed their expression (Fig. 3B). Consistently, immunoblotting analysis indicated that UCP1 protein was repressed by PA in a dose-dependent manner (Fig. 3C). Immunocytochemical analysis showed that high PA concentrations reduced UCP1 expression, whereas the amount of lipid droplets did not change significantly (Fig. 3D). In ciBAs derived from different HDF lines, Ch and PA were similarly required for the expression of *UCP1*, *CIDEA*, and *FABP4* in a dose-dependent manner (Supplementary Fig. S2A). Moreover, in adipocytes derived from immortalised human brown preadipocytes (hTERT A41hBAT-SVF) and adipose tissue-derived mesenchymal stem cells (AdMSCs), Ch and PA similarly regulated the expression, although PA affected *FABP4* expression more than in HDFs in a dose-dependent manner (Supplementary Fig. S2B,C). The response of *UCP1* expression to isoproterenol, a pan β-adrenergic receptor agonist, was not largely changed in PA-treated ciBAs at various concentrations (Supplementary Fig. S3). These results indicate that Ch and PA are required for adipogenesis and *UCP1* expression in multiple human brown adipocyte models and that PA downregulates *UCP1* expression at high concentrations.

High PA concentrations downregulated glycerol secretion and triglyceride accumulation (Fig. 3E,F). The activity of glycerol-3-phosphate dehydrogenase 1 (GPDH), a rate-limiting enzyme in triglyceride biosynthesis, was also reduced by PA at 16 μg/mL (Fig. 3G). The ratio of phosphorylated cAMP-response element binding protein (CREB) to total CREB protein was not altered by PA at 0.5 μg/mL (Fig. 3H). Although total CREB was reduced by PA at 16 μg/mL, the ratio was also reduced. In addition, the ratio of phosphorylated hormone-sensitive lipase (HLS), a rate-limiting enzyme in the first lipolytic process of triglycerides, was increased by low PA, whereas high PA reduced the ratio along with the reduction of total HSL levels. The cellular mitochondrial content was also reduced by high PA concentrations (Fig. 3I). These results suggest that PA treatment repressed UCP1 expression and cellular lipid metabolism in a dose-dependent manner.

### Pantothenic acid modulates mitochondrial respiration and glycolysis in a concentration-dependent manner

**Figure 4:**
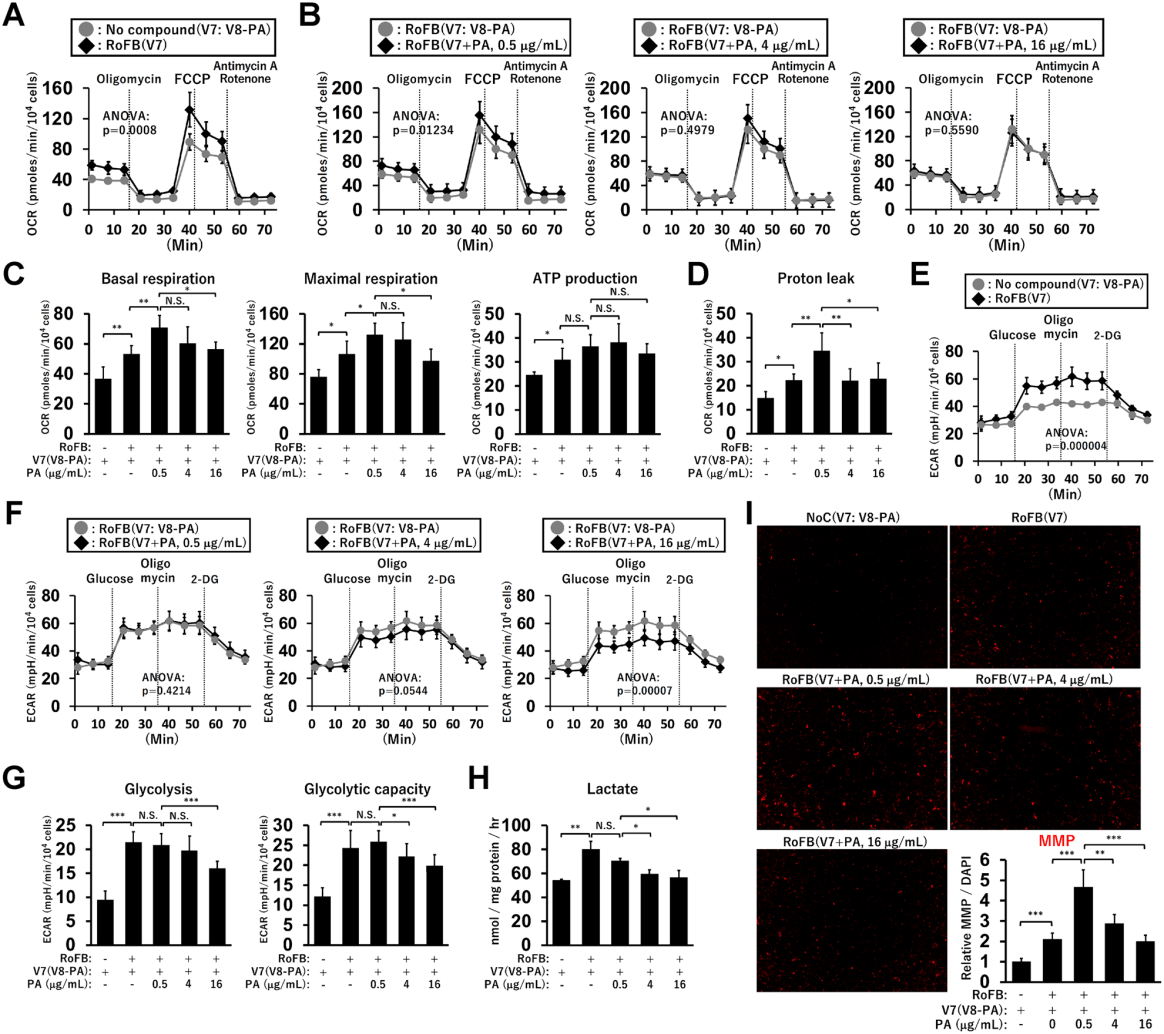
Concentration-dependent effects of PA on mitochondrial respiration and glycolysis. (**A**) Oxygen consumption rate (OCR) was measured using Seahorse XFe96 extracellular flux analyzer in control fibroblasts, NoC(V7) (grey circles), and ciBAs converted in the absence of PA, RoFB(V7) (black diamonds). Mitochondrial respiration inhibitors, oligomycin, FCCP, and antimycin A/rotenone, were added during the measurement, as indicated.* P* values were determined using two-way ANOVA. (**B**) OCR was compared between RoFB(V7) (grey circles) and either RoFB(V7 + PA, 0.5 μg/mL), RoFB(V7 + PA, 4 μg/mL), or RoFB(V7 + PA, 16 μg/mL) (black diamonds). *P* values were determined using two-way ANOVA. (**C,D**) OCR corresponding to basal respiration, maximal respiration, ATP production, and proton leak was compared. (**E**) Extracellular acidification rate (ECAR) was measured in control fibroblasts, NoC(V7) (grey circles), and ciBAs in the absence of PA, RoFB(V7) (black diamonds). Glucose, oligomycin, and 2-deoxyglucose (2-DG) were sequentially added during the measurement, as indicated. *P* values were determined using two-way ANOVA. (**F**) ECAR was also compared between the control ciBAs, RoFB(V7) (grey circles), and either RoFB(V7 + PA, 0.5 μg/mL), RoFB(V7 + PA, 4 μg/mL), or RoFB(V7 + PA, 16 μg/mL) (black diamonds). *P* values were determined using two-way ANOVA. (**G**) ECAR corresponding to glycolysis and glycolytic capacity was calculated. Data represent mean ± SD (*n* = 6–8). (**H**) Lactate secretion into culture supernatants was quantified in control fibroblasts and ciBAs treated with PA at various concentrations throughout the experiments. Data represent mean ± SD (*n* = 3). (**I**) Mitochondrial membrane potential (MMP) was evaluated by staining with the fluorescent probe, MT-1 dye. The area of the staining for the dye was quantified by ImageJ software. Data represent mean ± SD (*n* = 5). One-way ANOVA with Tukey’s multiple comparison tests: * *p* < 0.05, ** *p* < 0.01, *** *p* < 0.001, N.S.; not significant.

To examine the effects of PA on mitochondrial oxidation and glycolysis rates, ciBAs were analysed using a flux analyser in the presence or absence of PA (Fig. 4A). A low PA concentration (0.5 μg/mL) enhanced oxygen consumption rate (OCR) (Fig. 4B). However, the higher concentrations (4 and 16 μg/mL) reduced OCR, resembling those in PA-untreated ciBAs. Calculated OCR corresponding to basal respiration, maximal respiration, and ATP production was elevated in RoFB-induced and PA-untreated ciBAs (Fig. 4C). The basal and maximal respiration was increased by low PA, whereas high PA repressed the increase. Notably, similar to *UCP1* expression levels, OCR corresponding to proton leakage was altered by PA in a dose-dependent manner (Fig. 4D). Glycolysis rates were then evaluated by extracellular acidification rate (ECAR). ECAR in PA-untreated ciBAs was higher than that in control fibroblasts (Fig. 4E). ECAR was almost unchanged in ciBAs treated with low PA; however, high PA reduced it in a dose-dependent manner (Fig. 4F). Calculated ECAR corresponding to glycolysis and glycolytic capacity was decreased at high PA concentrations (Fig. 4G). PA also reduced lactate secretion in a dose-dependent manner (Fig. 4H). To further evaluate the regulation of mitochondrial energy status by PA, mitochondrial membrane potential (MMP) was quantified using a fluorescent probe (Fig. 4I). Similar to the OCR pattern, MMP was activated by low PA concentrations and deactivated by high PA concentrations. These results indicated that PA reduced mitochondrial respiration along with reduced glycolytic flux in a concentration-dependent manner.

### High concentration of pantothenic acid reverses the transcriptional activation of thermogenic and lipid metabolic genes

**Figure 5:**
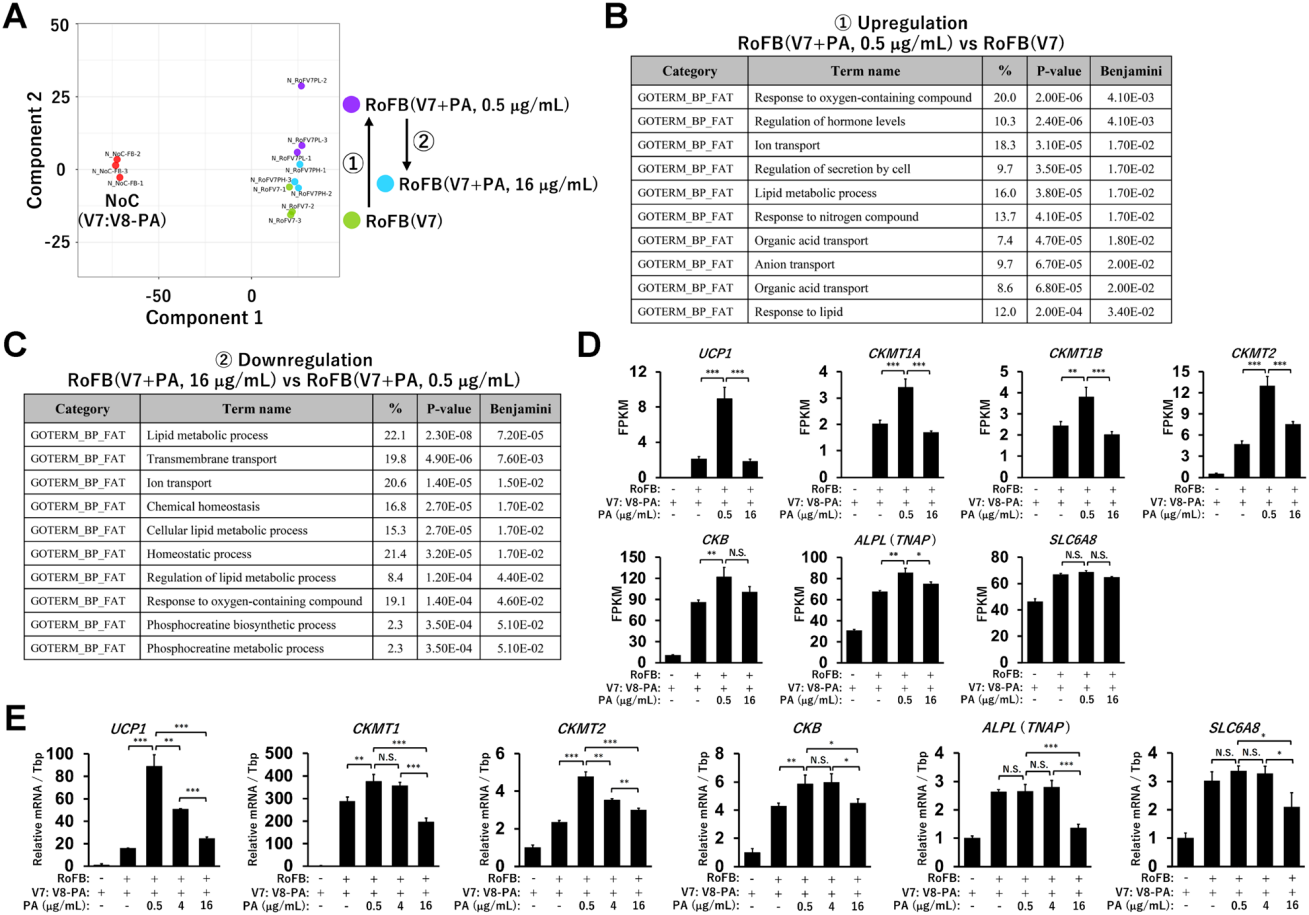
Comparison of the transcriptome in ciBAs treated with PA at low and high concentrations throughout the conversion. (**A**) Multidimensional scaling analysis graphically indicates the similarity and variability of the transcriptome in the control fibroblast, NoC(V7), ciBAs, RoFB(V7), and ciBAs treated with PA at low and high concentrations, RoFB(V7 + PA, 0.5 μg/mL) and RoFB(V7 + PA, 16 μg/mL). (**B**) Gene ontology (GO) enrichment analysis was performed in upregulated differentially expressed genes (DEGs) in RoFB(V7 + PA, 0.5 μg/mL) compared with RoFB(V7). The top 10 GO terms are represented in the category of biological process. (**C**) GO analysis was performed in downregulated DEGs in RoFB(V7 + PA, 16 μg/mL) compared with RoFB(V7 + PA, 0.5 μg/mL). (**D**) The FPKM values in the RNA-Seq results indicate the transcriptional levels of *UCP1*, *CKMT1A*, *CKMT1B*, *CKMT2*, *CKB*, *ALPL* (*TNAP*), *SLC6A8*, and *CKM* genes. (**E**) *UCP1*, *CKMT1*, *CKMT2*, *CKB*, *ALPL* (*TNAP*), and *SLC6A8* mRNA were measured by qRT-PCR analysis in ciBAs treated with PA as indicated. Data represent mean ± SD (n = 3). One-way ANOVA with Tukey’s multiple comparison tests: *P < 0.05, **P < 0.01, ***P < 0.001, N.S.; not significant.

To examine transcriptome changes regulated by PA, RNA-sequencing analysis (RNA-Seq) was performed. Multidimensional scaling analysis graphically showed a variability in total expression patterns in control fibroblasts and in PA-treated and PA-untreated ciBAs (Fig. 5A). As indicated by the component 2 axis in the graph, RoFB(V7 + PA, 0.5 μg/mL) was located higher than RoFB(V7), whereas RoFB(V7 + PA, 16 μg/mL) was returned to a location near RoFB(V7). The transcriptional changes between RoFB(V7 + PA, 0.5 μg/mL) and RoFB(V7 + PA, 16 μg/mL) likely reflected the reduced transcription of *UCP1* and other metabolic genes at high PA concentrations. A comparison of the RNA-Seq data between RoFB(V7) and RoFB(V7 + PA, 0.5 μg/mL) revealed 114 upregulated and 52 downregulated differentially expressed genes (DEGs) (Supplementary Fig. S4A). In addition, 53 upregulated and 57 downregulated DEGs were detected between RoFB(V7 + PA, 0.5 μg/mL) and RoFB(V7 + PA, 16 μg/mL) (Supplementary Fig. S4B). The smear and volcano plots showed that DEGs with over two-fold changes (FCs) were appropriately distributed with widespread counts per million (CPM) and P-values (Supplementary Fig. S4C,D). Gene ontology (GO) enrichment analysis indicated that DEGs upregulated in RoFB(V7 + PA, 0.5 μg/mL) could be categorised into functional groups, such as the response to oxygen-containing compound and the lipid metabolic process (Fig. 5B). Notably, the DEGs downregulated in RoFB(V7 + PA, 16 μg/mL) were categorised into similar functional groups that were upregulated by low PA levels (Fig. 5C). In addition, the downregulated DEGs included phosphocreatine (PCr) metabolic genes in the UCP1-independent thermogenic pathway in brown adipocytes. Accumulating evidence has suggested that the creatine-futile cycle contributes to adipose tissue thermogenesis^27,28^. RNA-Seq results indicated that PA regulated the transcription of PCr metabolic genes in a concentration-dependent manner, similar to the pattern observed for *UCP1* (Fig. 5D). The expression of muscle-specific creatine kinase (*CKM*) was negligible (Supplementary Fig. 5A). Quantitative real-time PCR (qRT-PCR) analysis confirmed that PA regulated the transcription of *CKMT1*, *CKMT2*, *CKB*, *ALPL* (*TNAP*), and *SLC6A8* genes (Fig. 5E). In addition, RNA-Seq results indicated that lipid metabolic genes were similarly regulated by PA in a dose-dependent manner (Supplementary Fig. S5B). In contrast, heatmaps showed that the transcription of major genes involved in glucose, fatty acid, and triglyceride metabolism were slightly different or largely consistent between PA treatments (Supplementary Fig. S5C,D). These results indicate that PA reversed the transcription of thermogenic and lipid metabolic genes activated by itself at high concentrations.

### Riboflavin represses thermogenic gene expression along with increased lipolysis

**Figure 6:**
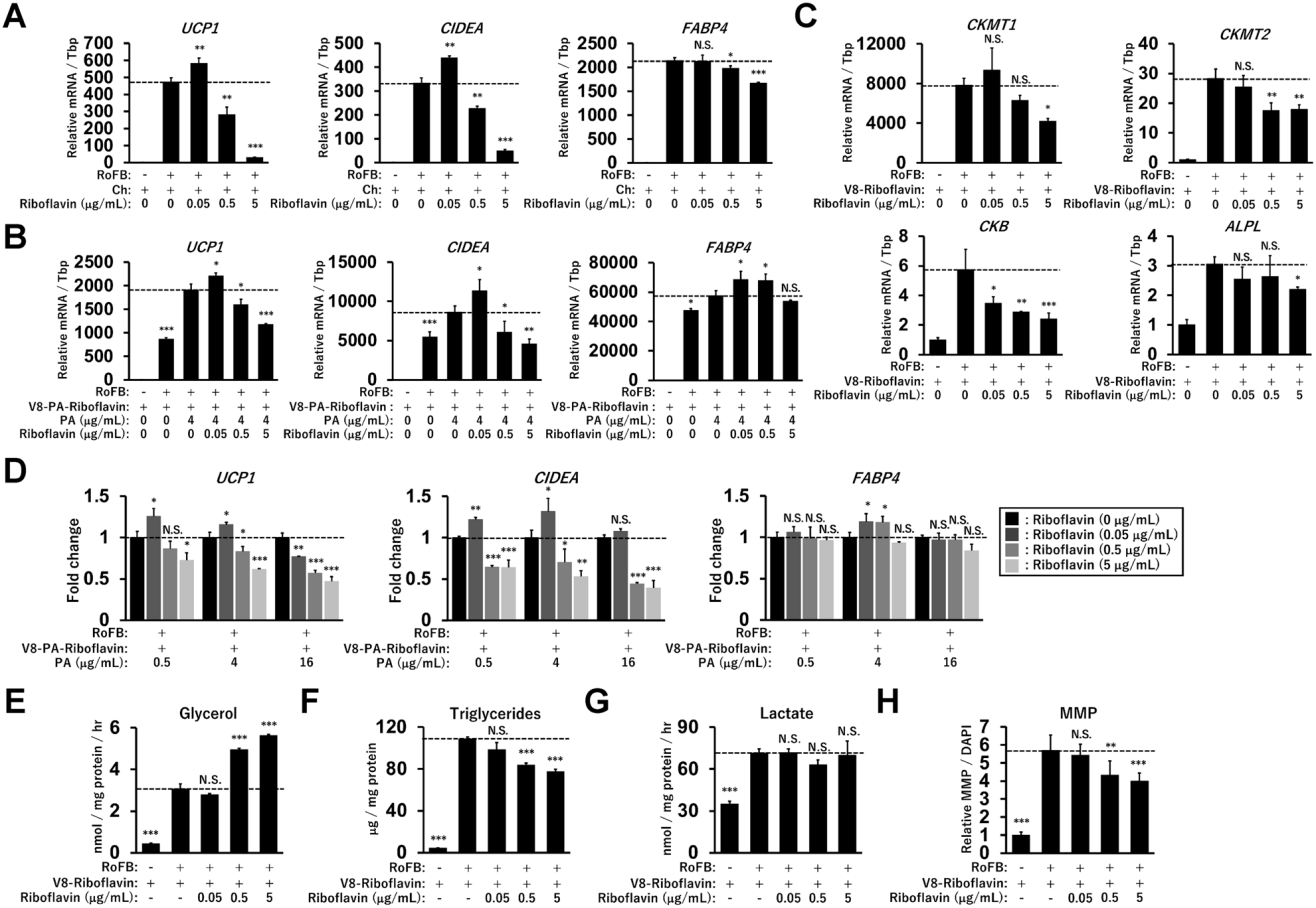
Effects of riboflavin on thermogenic gene expression and lipolysis in ciBAs. (**A**) The expression of *UCP1*, *CIDEA*, and *FABP4* was quantified by qRT-PCR analysis in ciBAs treated with Ch (4 μg/mL) and riboflavin at concentrations from 0.05 to 5 μg/mL throughout the experiments. (**B,C**) The expression of *UCP1*, *CIDEA*, *FABP4*, *CKMT1*, *CKMT2*, *CKB*, and *ALPL* was quantified by qRT-PCR analysis in ciBAs treated with riboflavin in the presence of the other vitamins. (**D**) The fold change of the expression was evaluated in ciBAs treated with the combination of PA and riboflavin at various concentrations, as indicated. (**E–H**) Glycerol secretion, triglyceride accumulation, lactate secretion, and MMP were measured in ciBAs treated with riboflavin at various concentrations. Data represent mean ± SD (n = 3). One-way ANOVA with Tukey’s multiple comparison tests: * *p* < 0.05, ** *p* < 0.01, *** *p* < 0.001, N.S.; not significant.

Figure 1 shows that riboflavin and thiamine may be involved in the regulation of *UCP1* expression. Because the effects of PA were concentration-dependent, the effects of riboflavin were examined at various concentrations. The addition of riboflavin at 0.05 μg/mL to the medium containing Ch only slightly enhanced the expression of *UCP1* and *CIDEA*, but not *FABP4* (Fig. 6A). However, riboflavin treatment at high concentrations (0.5 and 5 μg/mL) notably suppressed the expression. Under the medium including the other vitamins (V8-Riboflavin), riboflavin treatment also repressed the expression of *UCP1* and *CIDEA* at high concentrations (Fig. 6B). In addition, riboflavin reduced the expression of PCr metabolic genes such as *CKMT1*, *CKMT2*, *CKB*, and *ALPL* (Fig. 6C). Riboflavin and PA cooperatively repressed the transcription of *UCP1* and C*IDEA* in a dose-dependent manner (Fig. 6D). Riboflavin treatment at high concentrations promoted glycerol secretion (Fig. 6E), whereas triglyceride storage was slightly reduced (Fig. 6F). In contrast, lactate secretion was not altered by riboflavin treatment (Fig. 6G). MMP was reduced by riboflavin at high concentrations (Fig. 6H and Supplementary Fig. S6A). These results suggest that riboflavin treatment represses thermogenic gene expression and MMP along with activating triglyceride lipolysis in a dose-dependent manner.

### Thiamine slightly activates thermogenic gene expression along with reduced glycolysis

**Figure 7:**
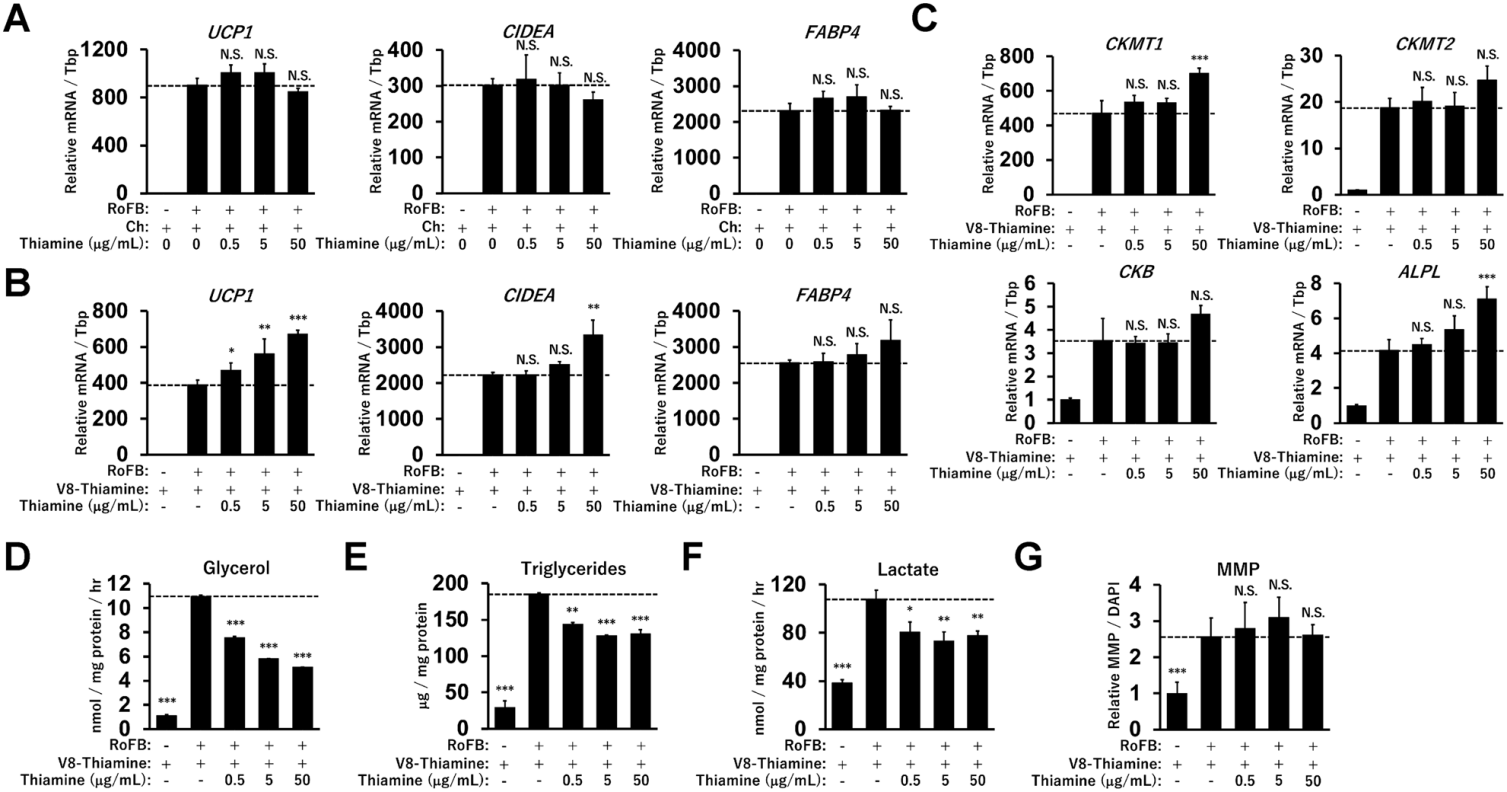
Effects of thiamine on thermogenic gene expression and glycolysis in ciBAs. (**A**) The expression of *UCP1*, *CIDEA*, and *FABP4* was quantified by qRT-PCR analysis in ciBAs treated with Ch (4 μg/mL) and thiamine at concentrations from 0.5 to 50 μg/mL throughout the experiments. (**B,C**) The expression of *UCP1*, *CIDEA*, *FABP4*, *CKMT1*, *CKMT2*, *CKB*, and *ALPL* was quantified by qRT-PCR analysis in ciBAs treated with thiamine in the presence of the other vitamins. (**D-G**) Glycerol secretion, triglyceride accumulation, lactate secretion, and MMP were measured in ciBAs treated with thiamine at various concentrations. Data represent mean ± SD (n = 3). One-way ANOVA with Tukey’s multiple comparison tests: * *p* < 0.05, ** *p* < 0.01, *** *p* < 0.001, N.S.; not significant.

Thiamine was then included in the Ch-only medium at various concentrations (Fig. 7A). The expression levels of *UCP1*, *CIDEA*, and *FABP4* were not significantly affected by thiamine treatment. In the presence of the other vitamins (V8-Thiamine), thiamine at high concentrations enhanced the expression of *UCP1*, *CIDEA*, and PCr metabolic genes (Fig. 7B,C). This treatment repressed glycerol secretion, triglyceride storage, and lactate secretion in a dose-dependent manner (Fig. 7D–F). MMP was largely unchanged by thiamine treatment (Fig. 7G and Supplementary Fig. S6B). These results indicate that thiamine is uniquely involved in the regulation of thermogenic gene expression through the metabolic changes in glucose and lipid metabolism.

## Discussion

**Figure 8:**
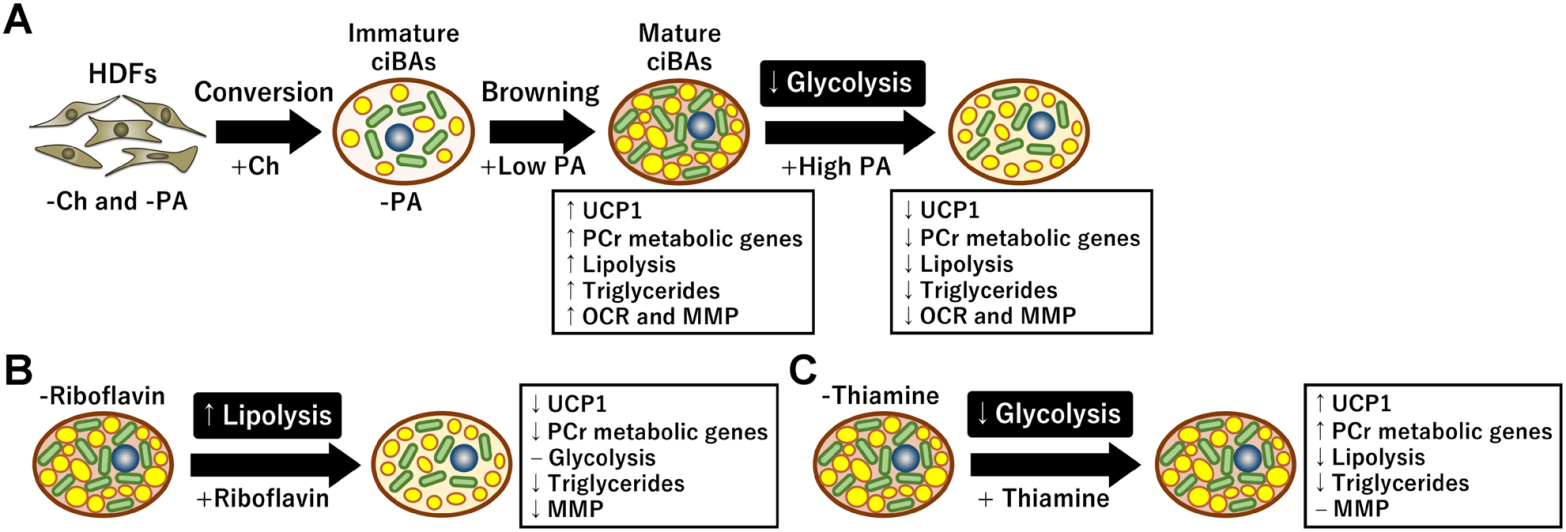
Schematic illustration of the role of choline (Ch), pantothenic acid (PA), riboflavin, and thiamine in the thermogenic and metabolic functions in ciBAs. (**A**) Ch is indispensable for adipocyte formation during the conversion of HDFs into ciBAs. The low concentration of PA is sufficient for Ch-induced immature ciBAs to enhance the expression of *UCP1* and phosphocreatine (PCr) metabolic genes, lipolysis, triglyceride accumulation, mitochondrial respiration, and MMP. However, treatment with PA at high concentrations represses PA-activated thermogenic expression and metabolic maturation along with reduced glycolysis. (**B**) Riboflavin treatment activates lipolysis, however, thermogenic gene expression, triglycerides, and MMP are repressed in a dose-dependent manner. (**C**) Thiamine treatment slightly activates thermogenic gene expression at high concentrations although glycolysis and lipolysis rates are reduced as a consequence.

In this study, the effects of vitamins on thermogenic gene expression and metabolic maturation in a human brown adipocyte model were identified. Modifying the components of the medium and eliminating serum enabled us to uncover the functions of specific vitamins under vitamin-free conditions. Among them, treatment with PA activated thermogenic gene expression, lipolysis, triglyceride storage, and mitochondrial respiration, suggesting that PA is a key vitamin that provides brown adipocyte-like characteristics (Fig. 8A). PA functions as cofactors for many enzymes involved in both catabolic and anabolic lipid metabolism, such as fatty acid β-oxidation (a catabolic process) and synthesis (an anabolic process). Therefore, PA can be considered supportive of metabolic maturation, including lipolysis and triglyceride accumulation, in Ch-induced adipocytes (Fig. 2F,G). However, this study showed that high PA concentrations reversed these effects and reduced glycolysis. This observation implies that high PA concentrations may support the conversion of pyruvate to acetyl-CoA via PDH to promote glycolysis to mitochondrial oxidative phosphorylation (Supplementary Fig. S7). This hypothesis is supported by the RNA-Seq results showing that the transcription of major metabolic genes involved in glycolysis, the TCA cycle, and fatty acid oxidation was largely unchanged in the high PA-treated ciBAs (Supplementary Fig. S5C,D). Notably, ciBAs treated with high PA showed decreased glycolysis and lactate secretion more than PA-untreated ciBAs; however, OCR did not change significantly (Fig. 4B,F–H). Anaplerosis promoted by high PA levels resulted in lower coupled and uncoupled mitochondrial respiration in ciBAs, which is associated with reduced adipocyte browning.

A PA deficiency is rare except in individuals with severe malnutrition^13^. Therefore, to clarify the functions of PA, a diet that either excludes PA or includes an inhibitor of the pantothenate kinase (PANK), a rate-limiting enzyme in CoA biosynthesis, was administered to animals. The PA-deficient diets decreased CoA levels to about 40% of normal levels in rat and duck livers, which was associated with a decreased ability to utilise pyruvate^29,30^. In addition, *Pank1*-deficient mice showed reduced hepatic CoA levels and an inhibited metabolic transition from glucose utilisation to fatty acid oxidation during fasting^31^. The same group showed that *Pank1*-deficiency in leptin-deficient obese mice ameliorated hyperglycemia and hyperinsulinemia^32^. Metabolic profiling by gas chromatography–mass spectrometry indicated that the liver of high-fat diet-induced obese mice elevated PA concentration by approximately three times and reduced the levels of many metabolites involved in glycolysis and the TCA cycle^33^, implying that high PA levels may lead to reduced glycolysis and mitochondrial respiration, similar to the findings of this study. In summary, these studies, including ours, suggest that reduced CoA levels, which occur due to PA-deficiency or PANK inhibition, may be beneficial for the prevention of obesity and related metabolic diseases.

B vitamins are generally maintained at low concentrations in the blood and are not stored in the body^34^. PA is transported from the intestinal lumen to the blood in its free form^35^. PA is then taken up by tissues and erythrocytes via passive diffusion^36^. The normal range of blood PA is typically 1.57–2.66 μM in healthy adults^37^, corresponding to 0.75–1.27 μg/mL of calcium pantothenate. In general, most PA is present in the form of CoA, whereas the amounts of free PA and acyl-carrier proteins are lower. A previous study using radiolabelled PA showed that it is highly concentrated in tissues, such as muscles (34.7 ± 2.5% of dose), liver (12.1 ± 2.2%), kidney (5.2 ± 0.7%), and colon (4.2 ± 0.3%)^38^. The evidence indicates that PA may accumulate more in brown adipose tissues than in the blood.

Ch was found to be indispensable for adipocyte generation during the conversion of HDFs to ciBAs (Fig. 8A). Ch-induced adipocytes enhanced triglyceride storage and lipolysis (Fig. 2F,G), which was related to the elevated expression of ATGL and CEBPA proteins involved in lipid metabolism (Fig. 2B,C). Ch is required for the biosynthesis of acetylcholine, phosphatidylcholine, and betaine^39^. Ch deficiency led to the development of hepatic steatosis in both humans and mice^40,41^. Consistently, Ch and betaine supplementation improved hypertriglyceridemia and hepatic steatosis^42^. However, the direct requirement of Ch for adipogenesis has not been examined in detail. This study provides novel insights into the role of Ch in adipocyte differentiation and function.

In cooperation with PA, riboflavin and thiamine are uniquely involved in thermogenic gene expression and the regulation of glucose and lipid metabolism (Supplementary Fig. S7). Riboflavin is the precursor of flavin mononucleotide (FMN) and flavin adenine dinucleotide (FAD), which function as redox cofactors in complex I and II in the electron transfer chain (ETC)^13^. Previous studies have shown that riboflavin-deficiency impairs mitochondrial oxygen consumption in brown adipose tissue in rat pups, but not in the liver^43,44^. This study indicates that riboflavin treatment enhanced lipolysis without altering glycolysis (Fig. 8B). However, riboflavin treatment repressed MMP and thermogenic gene expression. Both riboflavin and PA treatments cooperatively promoted adipocyte whitening in ciBAs likely through different metabolic pathways. This observation is supported by a recent epidemiological study in which the dietary intake of vitamin A, thiamine, riboflavin, cyanocobalamin, and vitamin D was negatively correlated with obesity in children and adolescents^45^. Further studies are required to determine the precise molecular mechanisms underlying the regulation of energy metabolism and thermogenic functions by riboflavin.

Thiamine pyrophosphate (TPP) also serves as a gatekeeper for PDH to connect glycolysis-derived pyruvate to the TCA cycle (Supplementary Fig. S7). Thiamine-deficient patients commonly develop lactic acidosis due to PDH inactivation^46^. A clinical study indicated that thiamine administration to the patients lowered blood lactate levels^47^. A recent report showed that a thiamine transporter, ThTr2, expressed in human brown adipocytes positively regulated cAMP-induced and PDH-mediated uncoupled mitochondrial respiration as well as *UCP1* transcription^48^. In addition, thiamine treatment suppressed the proliferation of breast cancer cells (MCF7) along with decreased glycolysis and increased PDH activity^49^. Another recent report on the differentiation of human subcutaneous and deep neck-derived preadipocytes showed that an excess amount of thiamine increased OCR and the transcription of thermogenic genes including *UCP1*^50^. Similar to these reports, our findings indicated that thiamine treatment increased thermogenic gene expression and reduced glycolysis (Fig. 8C). These results suggest that among B vitamins, riboflavin and thiamine contribute differentially to the regulation of thermogenic gene expression and cellular energy metabolism in human brown adipocytes.

Transcriptome analysis indicated that the transcription of not only UCP1 but also PCr metabolic genes was controlled in parallel by PA in a dose-dependent manner in ciBAs. The creatine-phosphocreatine futile cycle is one of the UCP1-independent thermogenic pathways in brown adipocytes. CKB is a major creatine kinase isoenzyme in mouse and human brown adipocytes and its expression was induced by cold exposure and a β3-adrenergic receptor agonist^51^. Consistently, the fragments per kilobase of transcript per million mapped sequence reads (FPKM) values in the RNA-Seq analysis indicated that *CKB* was more abundantly expressed than *CKMT1* and *CKMT2* in ciBAs. Brown adipocyte-specific *CKB* knockout mice showed reduced mitochondrial respiration in primary brown adipocytes and increased susceptibility to diet-induced obesity. Another recent study showed that the inducible adipocyte-selective deletion of either UCP1 or CKB was still tolerant to hypothermia^52^. However, the co-deletion exacerbated cold intolerance, suggesting that they both contributed to thermogenesis in brown adipocytes in parallel. The knockdown of CKMT1 also reduced mitochondrial respiration and induced UCP1 expression in human brown adipocytes^53^. Moreover, the single nucleotide polymorphisms in CKB and CKMT1B were associated with BMI^54^, indicating that CKB and CKMT1 are involved in the pathogenesis of obesity in vivo. Thus, the evidence suggests that the downregulation of CKB and CKMTs by high concentrations of PA and riboflavin may be associated with reduced mitochondrial respiration and MMP through the futile creatine cycle. Further studies are required to reveal the precise molecular mechanism underlying how cellular energy metabolism regulated by PA and riboflavin is associated with the transcriptional regulation of these thermogenic genes. Furthermore, to precisely quantify UCP1-independent thermogenic functions through the transcriptional regulation of CKB by these vitamins, comparing OCRs corresponding to proton leak using UCP1-deficient brown adipocytes is required.

Both vitamin deficiency and excessive vitamin supplementation are associated with the development of obesity and metabolic diseases^55^. Previous reports have indicated that several vitamins, including PA, riboflavin, and thiamine, are negatively correlated with BMI and obesity^17,45,56^. Accumulating scientific evidence on the effects of vitamins is supportive of developing dietary interventions for the long-term management of obesity in combination with pharmacological and physical therapies. This study revealed that choline and specific B vitamins were closely associated with brown adipogenesis and adipocyte browning in a concentration-dependent manner, respectively. However, excessive intake of PA and riboflavin may not always prevent obesity and metabolic diseases through thermogenic functions in brown adipocytes. Mechanistic insights into regulating adipocyte browning by PA and other B vitamins remains unclear. In addition, the detailed mechanism underlying why low PA and high PA indicates contradictory effects has not been sufficiently elucidated. Overnutrition may adversely affect metabolic conditions, similar to the observation that the prevalence and activity of human brown adipocytes are inversely correlated with BMI^10^. Metabolome analysis using radio-labelled PA and other B vitamins could help to uncover the detailed mechanism. Analyses using immortalized human brown adipocytes deficient of key metabolic enzymes or transcriptional factors are also required in the future. The manipulation of the mass and activity of human brown adipocytes via the control of vitamin intake may foster a safe and effective nutritional strategy for counteracting obesity and metabolic diseases. The vitamin-free cell culture system used in this study provides insights into the direct effects of vitamins on thermogenic functions through cellular energy metabolism in human brown adipocytes.

## Methods

### Cell culture

Fibroblasts derived from a human subject aged 38 years (HDF38) (PromoCell, Heidelberg, Germany) were used in this study^24^. Approximately 1.5 × 10^5^ cells were seeded on a 35-mm dish with high-glucose Dulbecco's Modified Eagle Medium (DMEM) (11995-065, Gibco, MA, USA) supplemented with 10% fetal bovine serum (FBS) (HyClone, UT, USA) and penicillin/streptomycin (Gibco). After reaching 80–90% confluence, the direct conversion into ciBAs was started with the serum-free brown adipogenic medium (SFBAM) prepared from high-glucose DMEM (11995-065, Gibco) supplemented with linoleic acid- and oleic acid-albumin (L9655-5ML, Sigma-Aldrich, MO, USA), 3,3′,5 triiodothyronine (T3) (Sigma-Aldrich), dexamethasone (FUJIFILM Wako, Osaka, Japan), 3-isobutyl-1-methylxanthine (IBMX) (FUJIFILM Wako), human recombinant insulin (FUJIFILM Wako), L-ascorbic acid-2-phosphate (Sigma-Aldrich), and penicillin/streptomycin (Gibco). HDF38 cells were incubated with SFBAM either with or without the chemical combination RoFB, which consisted of 1 μM rosiglitazone (FUJIFILM Wako), 7.5 μM forskolin (FUJIFILM Wako), and 20 ng/mL human recombinant BMP7 (FUJIFILM Wako) for 3 weeks unless otherwise indicated^23^. Other lines of human dermal fibroblasts (HDF35 and HDF54) were also purchased from PromoCell^24^. The immortalised preadipocyte cell line isolated from human deep neck fat tissue (hTERT A41hBAT-SVF) was purchased from the American Type Culture Collection (CRL-3385, ATCC, VA, USA)^26^. MSCs derived from the adipose tissue of human subjects aged 38 (AdMSC38) and 51 (AdMSC51) years were purchased from TaKaRa Bio (C-12977, TaKaRa Bio, Shiga, Japan) and cultured in Mesenchymal Stem Cell Growth Medium 2 (TaKaRa Bio)^24^. These cells were differentiated into brown-like adipocytes in the same manner as ciBAs. These commercial human cells have been approved for in vitro research use only. All cell culture experimental procedures were conducted in accordance with the general guidelines of the Kyoto Prefectural University of Medicine.

Custom-made DMEM without vitamins and vitamin-like compounds was obtained from the Basal/Classical Medium Manufacturing Service at GMEP Inc. (Fukuoka, Japan). HDFs were converted into adipocytes using SFBAM prepared from the vitamin-free DMEM. Choline chloride (034-13132, FUJIFILM Wako), calcium ( +)-pantothenate (031-14161, FUJIFILM Wako), folic acid (062-01801, FUJIFILM Wako), nicotinamide (N0636, Sigma-Aldrich), pyridoxine hydrochloride (165-05401, FUJIFILM Wako), riboflavin (180-00171, FUJIFILM Wako), thiamine hydrochloride (203-00851, FUJIFILM Wako), and myo-inositol (092-00282, FUJIFILM Wako) were separately added to the vitamin-free SFBAM. Unless otherwise indicated, these vitamins were supplied throughout the conversion of HDFs into ciBAs. Custom-made DMEM without either the combination of Ch and PA or the combination of PA, riboflavin, and thiamine was also used in this study.

### qRT-PCR

qRT-PCR was performed as previously described^25^. In short, total RNA was extracted from control fibroblasts, ciBAs, and differentiated adipocytes using the FastGene RNA Basic Kit (Nippon Genetics, Tokyo, Japan). Reverse transcription was conducted using ReverTra Ace qPCR RT Master Mix with gDNA Remover (TOYOBO, Osaka, Japan). The qRT-PCR analysis was performed using Power SYBR Green PCR Master Mix (Applied Biosystems, MA, USA). All the results were normalised to *TBP* mRNA levels. Primer sequences used for qRT-PCR are listed in Supplementary Table S1. Unless otherwise indicated, the average of three biological replicates was calculated.

### Immunoblotting

For immunoblot analysis, total protein was extracted from control fibroblasts and ciBAs using RIPA buffer (FUJIFILM Wako) containing a phosphatase inhibitor cocktail (FUJIFILM Wako) and a protease inhibitor cocktail (FUJIFILM Wako). The extracted proteins were subjected to sodium dodecyl sulfate–polyacrylamide gel electrophoresis using a 10% gel concentration and transferred to a polyvinylidene fluoride membrane (Thermo Fisher Scientific, MA, USA). The membranes were blocked with 3% skim milk followed by incubation with antibodies against UCP1 (MAB6158, R&D Systems, MN, USA), ATGL (#2138, Cell Signaling Technology, MA, USA), CEBPA (#8178, Cell Signaling Technology), β-Actin (A5316, Sigma-Aldrich), P-CREB (#9198, Cell Signaling Technology), CREB (#9197, Cell Signaling Technology), P-HSL (#18381, Cell Signaling Technology), and HSL (#4107, Cell Signaling Technology) at 4 °C overnight. Membranes were then incubated with either horseradish peroxidase (HRP)-conjugated anti-rabbit or anti-mouse secondary antibodies (Cell Signaling Technology) for 1 h at approximately 25 °C (room temperature). Immunoreactive bands were detected using Immobilon Western Chemiluminescent HRP Substrate (Merck Millipore, Darmstadt, Germany). The intensity of each band was quantified by densitometry using the ImageJ software version 1.52 (National Institutes of Health) (https://imagej.net/ij/index.html). The experiments were performed independently at least twice.

### Immunocytochemistry

ciBAs were incubated with 1 µM Lipi-Red (Dojindo, Kumamoto, Japan) for 30 min at 37 °C in 5% CO_2_, according to the manufacturer’s instructions. The cells were then fixed with 4% paraformaldehyde for 10 min. After washing with phosphate-buffered saline (PBS), the cells were incubated in PBS containing 0.1% Triton X-100 for 5 min. After incubation, they were blocked with PBS containing 3% skim milk for 1 h at approximately 25 °C and incubated again with UCP1 antibody (ab10983, Abcam, Cambridge, UK) at 1/1000 dilution overnight at 4 °C. The cells were then incubated with Alexa Fluor 488 donkey anti-rabbit IgG (Invitrogen, CA, USA) for 1 h at approximately 25 °C. Subsequently, cell nuclei were stained with 4′,6-diamidino-2-phenylindole (DAPI) solution (Dojindo). All images were obtained using a BZ-X710-All-in-One Fluorescence Microscope (Keyence, Osaka, Japan) with a 20X objective lens (CFI Plan Fluor 20X, Nikon, Tokyo, Japan). All scale bars represent 200 μm. The areas of Lipi-Red and UCP1 staining were quantified using ImageJ software from at least five different optical sections.

### Measurement of OCR and ECAR

To measure OCR, HDF38 cells were seeded on a 96-well plate and converted to ciBAs in the presence or absence of PA at various concentrations for 3 weeks. Before the measurement, the cells were washed and incubated with non-buffered DMEM supplemented with 25 mM glucose, 2 mM glutamine, and 1 mM pyruvate at 37 °C in a non-CO_2_ incubator for 1 h. OCR was then measured using the Seahorse XF96 Extracellular Flux Analyzer (Seahorse Bioscience Inc., MA, USA) according to the manufacturer’s instructions. Oligomycin, carbonyl cyanide 4-(trifluoromethoxy)phenylhydrazone (FCCP), and antimycin A/rotenone were added into each well using an injection apparatus at final concentrations of 2 μM, 0.3 μM, and 0.5 μM, respectively. The OCR corresponding to each mitochondrial parameter was determined by subtracting antimycin A/rotenone-insensitive OCR values from the other OCR values. To measure glycolytic flux, the cells were incubated in non-buffered DMEM without glucose and pyruvate for 1 h at 37 °C in a non-CO2 incubator. ECAR was measured using the Flux Analyze by adding glucose (Sigma-Aldrich), oligomycin (Sigma-Aldrich), 2-deoxy-D-glycose (Tokyo Chemical Industry) via an injection apparatus during the measurement to final concentrations of 10 mM, 5 μM, and 50 mM. The ECAR corresponding to glycolysis and glycolytic capacity were determined by subtracting 2-DG-insensitive ECAR values from the other ECAR values before and after oligomycin treatment, respectively. The average of six to eight biological replicates was calculated.

### Measurement of glycerol, triglycerides, lactate and GPDH activity

Cell culture supernatants and cell lysates were collected from control fibroblasts and ciBAs. Free glycerol content, lactate secretion, and GPDH activity were measured using the Free Glycerol Assay Kit (ab65337, Abcam), Lactate Assay Kit-WST (L256, Dojindo), and GPDH Assay kit (AK01, Cosmo Bio Co., Ltd., Tokyo, Japan), respectively, according to the manufacturer’s instructions. For measurement of cellular triglyceride contents, lipids extracted from the cells using 5% NP-40/ddH_2_O solution were quantified using the Triglyceride Assay kit (Ab65336, Abcam). All the experiments were performed in triplicates. The levels of glycerol, triglycerides, and GPDH activity were normalised to protein levels in each cell culture.

### Mitochondrial content and membrane potential

Mitochondria in ciBAs were stained by MitoTracker® Red CM-H_2_XRos (Thermo Fisher Scientific, DE, USA). In brief, the cells were pre-incubated with MitoTracker for 30 min at 37 °C in a 5% CO_2_ before the cells were fixed with 4% paraformaldehyde for 10 min. MMP in control fibroblasts and ciBAs were stained using the MT-1 MitoMP Detection kit (MT13, Dojindo). The cells were treated with the MT-1 dye for 30 min at 37 °C in a 5% CO_2_ incubator, according to the manufacturer’s instruction. All images were captured using a BZ-X710-All-in-One Fluorescence Microscope. The area of staining was quantified from at least five different optical sections using ImageJ software.

### RNA-Sequencing (RNA-Seq)

RNA-Seq analysis was performed as described previously^24^. Briefly, the library was prepared using the TruSeq stranded mRNA LT Sample Prep Kit (Illumina, CA, USA), following the manufacturer’s low sample (LS) instructions. Paired-end sequencing (100 bp) was performed by the NovaSeq 6000 System (Illumina). Trimmed reads were mapped to a reference genome (NCBI GRCh38) with HISAT2. After transcript assembly, the abundance of genes/transcripts was calculated from the read counts and normalised as FPKM. For the identification of DEGs, statistical analysis was performed using FC and the exact test using edgeR per comparison pair. Significant results satisfying the conditions of |FC|≥ 2 and the exact test p-value < 0.05 were selected. If more than one read count value was zero, it was excluded from the analysis.

### Data analysis

Heat maps were generated using Heatmapper (http://www.heatmapper.ca/)^57^. Each row represents a gene and each column represents the z-scored FPKM of each sample. The green and magenta gradients represent low and high gene expression, respectively. Gene ontology enrichment analysis was performed by DAVID Bioinformatics Resources 6.8 (https://david.ncifcrf.gov/)^58^.

### Statistical analyses

All the results are presented as the mean ± standard deviation (SD). Statistical analyses were performed by a two-tailed Student’s *t*-test between two independent groups in the Excel (Microsoft, WA, USA) program. One-way ANOVA with Tukey’s multiple comparison tests was performed between multiple independent groups using an R-based statistical software, EZR version 1.65 (Saitama Medical Center, Jichi Medical University, Saitama, Japan) (https://www.jichi.ac.jp/saitama-sct/SaitamaHP.files/statmedEN.html), unless otherwise indicated^59^. Two-way ANOVA was also used for multiple comparison, as described in Fig. 4. Statistical significance was defined as p < 0.05.

### Supplementary Information

Supplementary Information.

## Data Availability

The RNA-sequencing data have been deposited in the DNA Data Bank of Japan (DDBJ) Sequenced Read Archive (https://www.ddbj.nig.ac.jp/dra/index-e.html) under the accession numbers DRR540151–DRR540159.
