# Supplementary material for: Functional roles of pantothenic acid, riboflavin, thiamine, and choline in adipocyte browning in chemically induced human brown adipocytes
Source: Sci Rep. 2024 Aug 6;14:18252. doi: 10.1038/s41598-024-69364-w (PMC11303702; doi:10.1038/s41598-024-69364-w)
Supplement: Supplementary file 1 — Supplementary Information. [file 41598_2024_69364_MOESM1_ESM.pdf]

Functional roles of pantothenic acid, riboflavin, thiamine, and choline in adipocyte browning in chemically induced human brown adipocytes.

Yukimasa Takeda* and Ping Dai*

Department of Cellular Regenerative Medicine, Graduate School of Medical Science, Kyoto Prefectural University of Medicine, 465 Kajii-cho, Kawaramachi-Hirokoji, Kamigyo-ku, Kyoto 602-8566, Japan

Supplementary Information

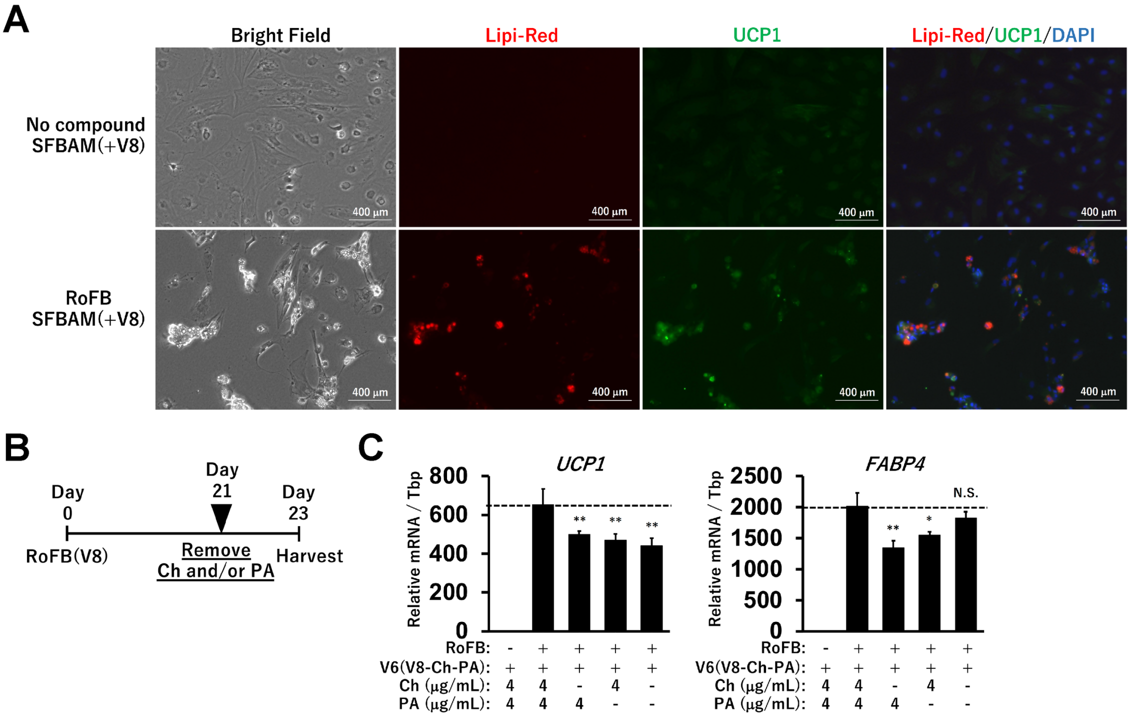

Supplementary Figure S1. Short term effects of the deficiency of choline and pantothenic acid for a short period on *UCP1* expression. (**A**) Immunocytochemical images of bright field, lipid droplets stained by Lipi-Red (red), UCP1 expression (green), and merged image indicated that brown-like adipocytes were successfully generated from primary HDFs by treatment with the chemical cocktail, RoFB. (**B**) The illustration shows the timing of either Ch or PA removal on day 21 after starting the conversion of HDF into ciBAs with the chemical cocktail, RoFB, including the eight vitamins (V8) on day 0. (**C**) The expression of *UCP1* and *FABP4* was analyzed in the ciBAs cultured without either Ch, PA, or both for the last 2 days. Data represent mean ± SD ($n = 3$). One-way ANOVA with Tukey's multiple comparison tests: * $p < 0.05$, ** $p < 0.01$, N.S.; not significant.

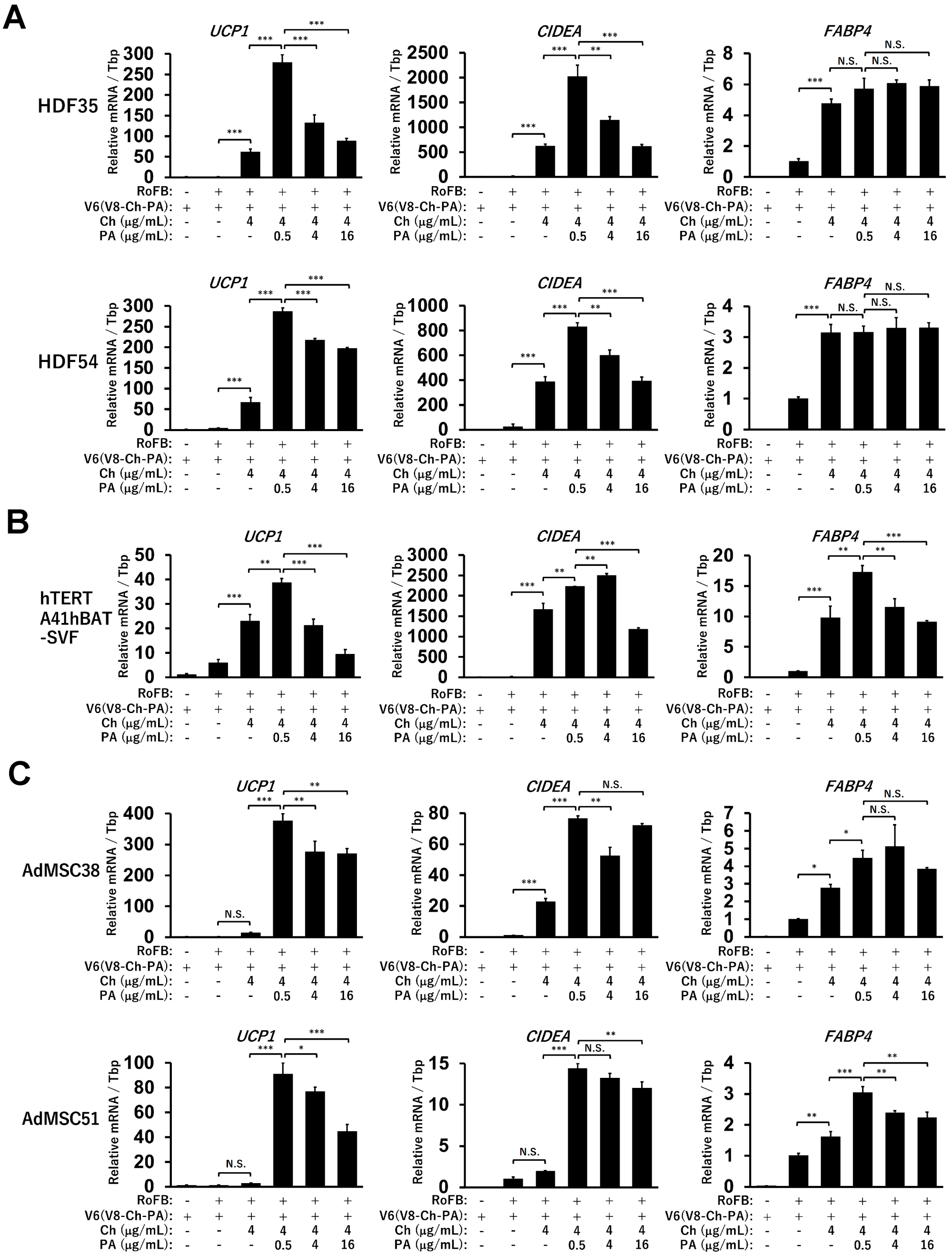

Supplementary Figure S2. Effects of choline and pantothenic acid on the expression of *UCP1*, *CIDEA*, and *FABP4* in the models of human brown adipocytes. (**A-C**) The expression was analyzed by qRT-PCR in ciBAs derived from other lines of HDFs (HDF35 and HDF54) and adipocytes derived from immortalized human brown preadipocytes (hTERT A41hBAT-SVF), and AdMSCs (AdMSC38 and AdMSC51). Data represent mean ± SD (n = 3). One-way ANOVA with Tukey's multiple comparison tests: * $p < 0.05$, ** $p < 0.01$, ***P<0.001, N.S.; not significant.

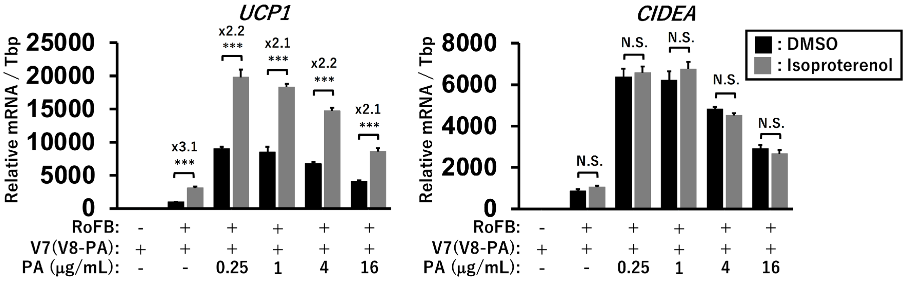

Supplementary Figure S3. The response to a β-adrenergic receptor agonist, isoproterenol, was evaluated by the fold change of *UCP1* and *CIDEA* expression between ciBAs treated with either DMSO or isoproterenol for 6 h. Data represent mean ± SD (n = 3). Student's t-test: *** $p < 0.001$, N.S.; not significant.

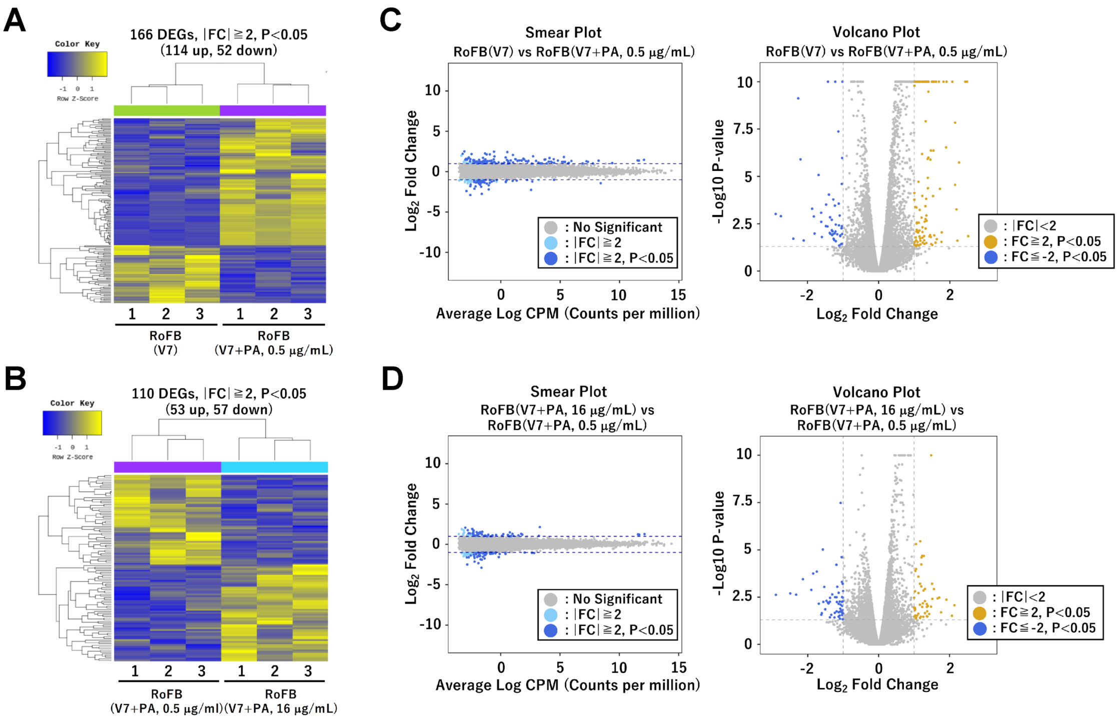

Supplementary Figure S4. Transcriptome analysis in ciBAs treated with pantothenic acid at low and high concentrations throughout the conversion. (**A**) Heat map and hierarchical clustering analysis represent 166 differentially expressed genes (DEGs) (|fold change (FC)| ≥ 2, p < 0.05) between control ciBAs, RoFB(V7), and ciBAs treated with PA at a low concentration, RoFB(V7+PA, 0.5 μg/mL). (**B**) Heat map and hierarchical clustering analysis represent 110 DEGs between ciBAs treated with PA at low and high concentrations, RoFB(V7+PA, 0.5 μg/mL) and RoFB(V7+PA, 16 μg/mL). (**C,D**) Smear and Volcano plots indicate logarithmic FC, P-value, and CPM (counts per million) between RoFB(V7) and RoFB(V7+PA, 0.5 μg/mL) and between RoFB(V7+PA, 16 μg/mL) and RoFB(V7+PA, 0.5 μg/mL).

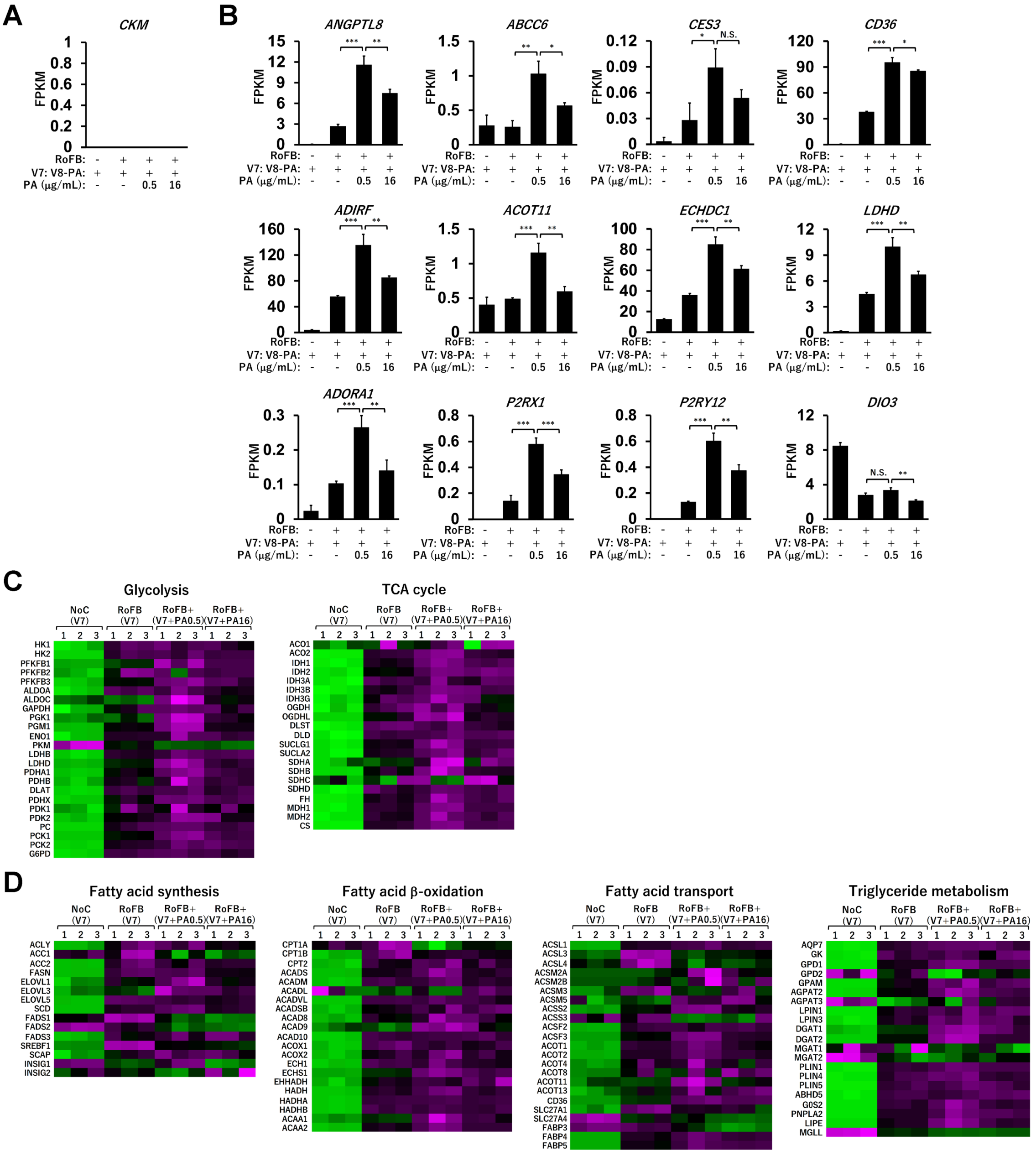

Supplementary Figure S5. Heat map analysis for the transcriptome in ciBAs treated with pantothenic acid at low and high concentrations throughout the conversion. (**A**) The FPKM values of CKM gene were obtained from the RNA-Seq results. (**B**) The FPKM values of lipid metabolic genes, such as *ANGPTL8*, *ABCC6*, *CES3*, *CD36*, *ADIRF*, *ACOT11*, *ECHDC1*, *LDHD*, *ADORA1*, *P2RX1*, *P2RY12*, and *DIO3* were shown. Data represent mean ± SD ($n = 3$). One-way ANOVA with Tukey's multiple comparison tests:

* $p < 0.05$, ** $p < 0.01$, ***$P<0.001$, N.S.; not significant. (**C,D**) Heat maps are shown in PA-treated ciBAs at low (0.5 µg/mL) and high (16 µg/mL) concentrations in functional groups such as the glycolysis pathway, TCA cycle, fatty acid synthesis, β-oxidation, transport, and triglyceride metabolism. The color scale shows z-scored fragments per kilobase of transcript per million mapped sequence reads (FPKM) representing mRNA levels of each gene in green (lower expression) and magenta (higher expression).

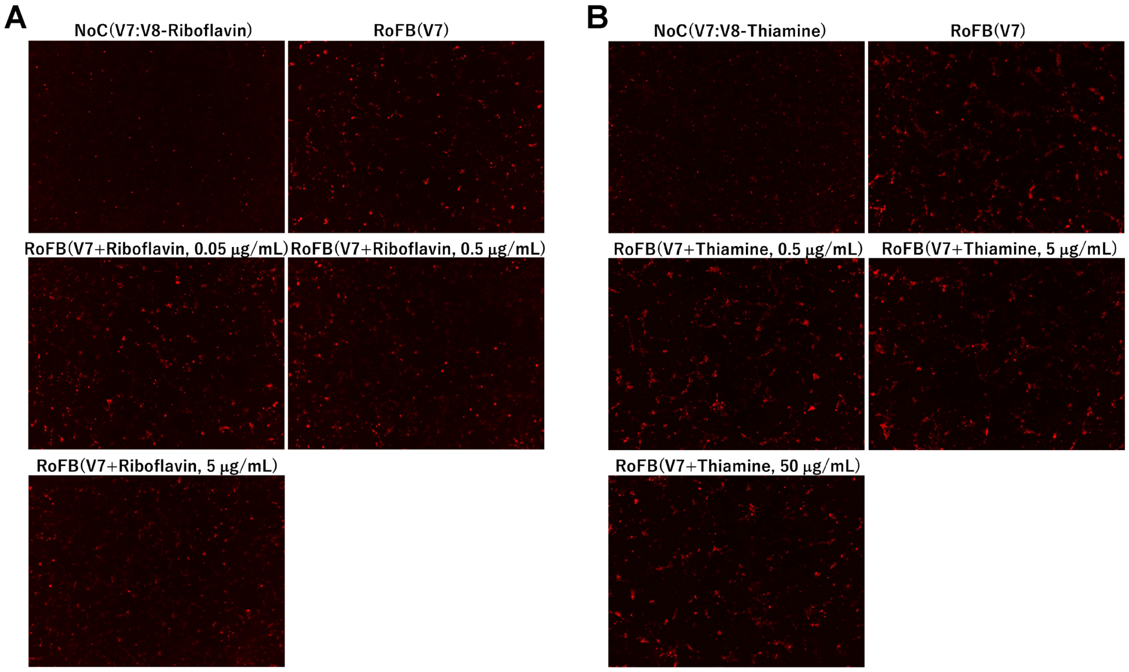

Supplementary Figure S6. Representative images of the staining of MT-1 dye for the quantification of MMP in ciBAs treated with either riboflavin (**A**) or thiamine (**B**) at indicated concentrations throughout the conversion.

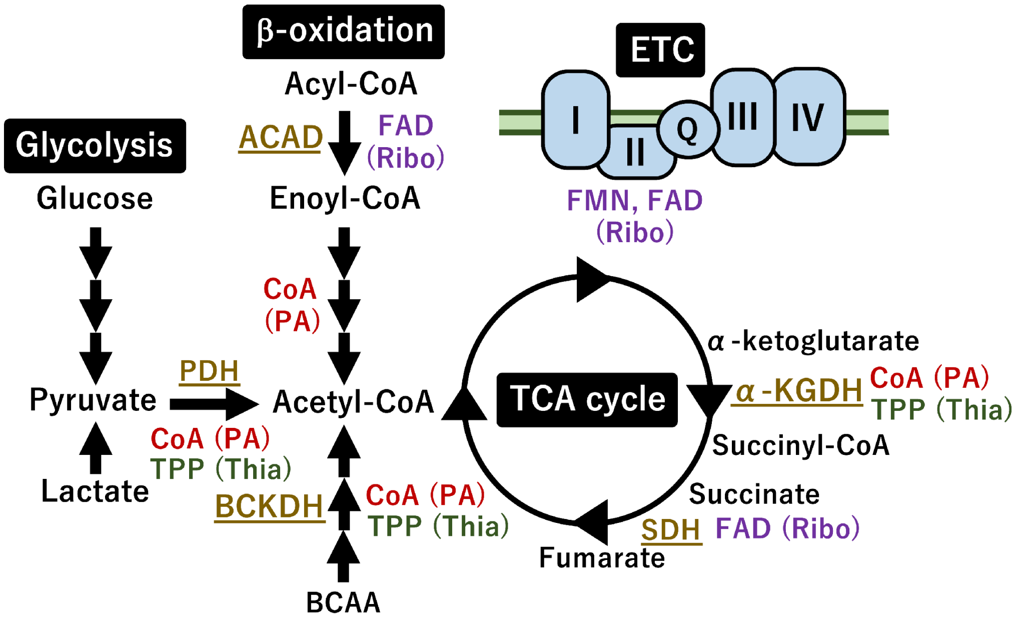

Supplementary Figure S7. Regulation of cellular energy metabolism by cofactors derived from pantothenic acid (PA), riboflavin (Ribo), and thiamine (Thia). Coenzyme A (CoA) and thiamine pyrophosphate (TPP) synthesized from pantothenic acid and thiamine, respectively, function as cofactors for pyruvate dehydrogenase (PDH), alpha-ketoglutarate dehydrogenase (α-KGDH), and branched-chain α-keto acid dehydrogenase (BCKDH). Flavin mononucleotide (FMN) and flavin adenine dinucleotide (FAD) synthesized from riboflavin are required for succinate dehydrogenase (SDH) in the TCA cycle, complex I and II in the electron transfer chain (ETC), and acyl-CoA dehydrogenase (ACAD) in the β-oxidation. This study suggests that these cofactors uniquely control cellular glycolysis and lipolysis pathways, which contribute to thermogenic gene expression and mitochondrial energy status in a human brown adipocyte model.

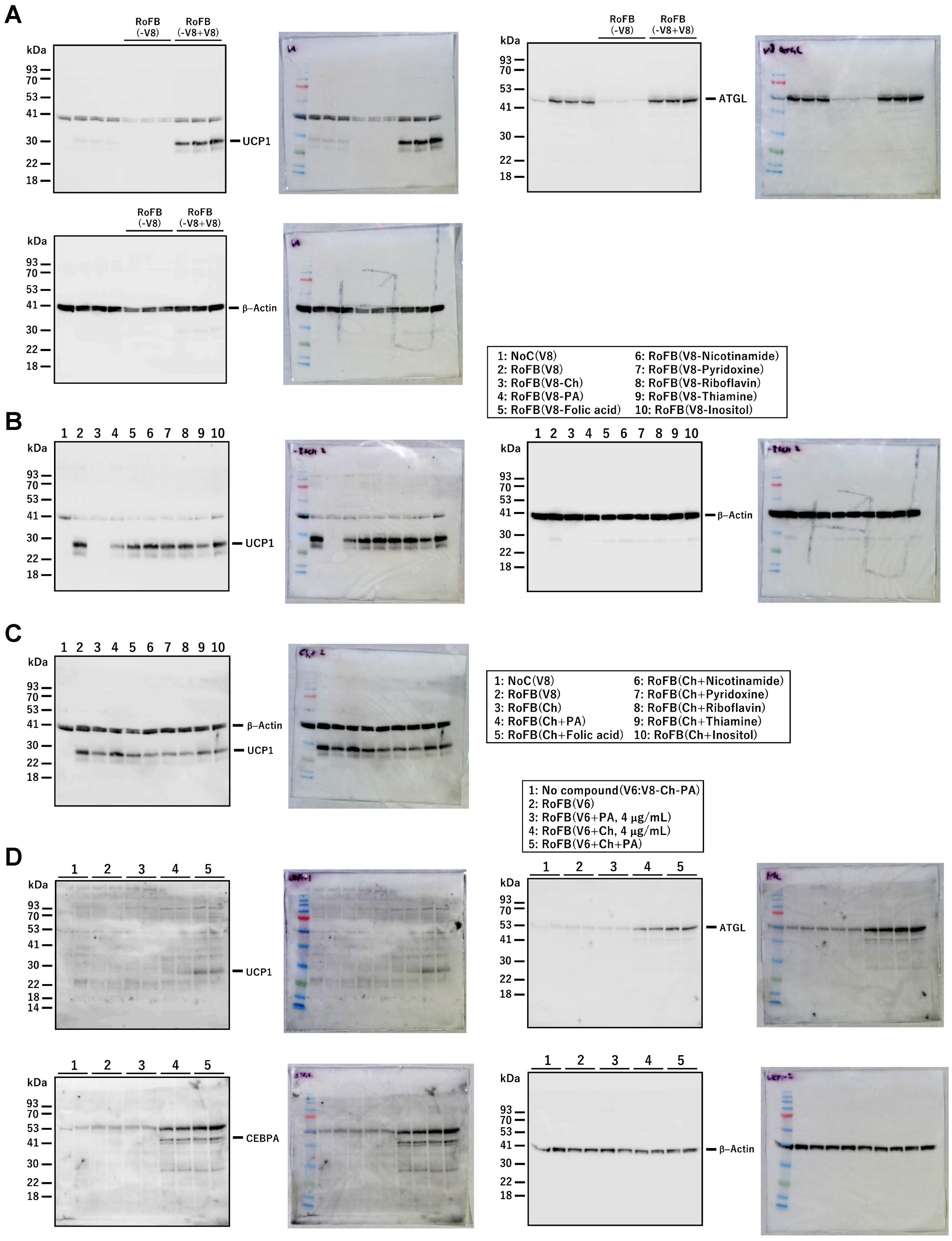
A
RoFB (-V8)
RoFB (-V8+V8)
kDa
93
70
53
41
30
22
18
UCP1
ATGL
β-Actin
1: NoC(V8)
2: RoFB(V8)
3: RoFB(V8-Ch)
4: RoFB(V8-PA)
5: RoFB(V8-Folic acid)
6: RoFB(V8-Nicotinamide)
7: RoFB(V8-Pyridoxine)
8: RoFB(V8-Riboflavin)
9: RoFB(V8-Thiamine)
10: RoFB(V8-Inositol)
B
1 2 3 4 5 6 7 8 9 10
UCP1
β-Actin
C
1 2 3 4 5 6 7 8 9 10
β-Actin
UCP1
1: NoC(V8)
2: RoFB(V8)
3: RoFB(Ch)
4: RoFB(Ch+PA)
5: RoFB(Ch+Folic acid)
6: RoFB(Ch+Nicotinamide)
7: RoFB(Ch+Pyridoxine)
8: RoFB(Ch+Riboflavin)
9: RoFB(Ch+Thiamine)
10: RoFB(Ch+Inositol)
1: No compound(V6:V8-Ch-PA)
2: RoFB(V6)
3: RoFB(V6+PA, 4 μg/mL)
4: RoFB(V6+Ch, 4 μg/mL)
5: RoFB(V6+Ch+PA)
D
1 2 3 4 5
14
UCP1
ATGL
CEBPA
β-Actin

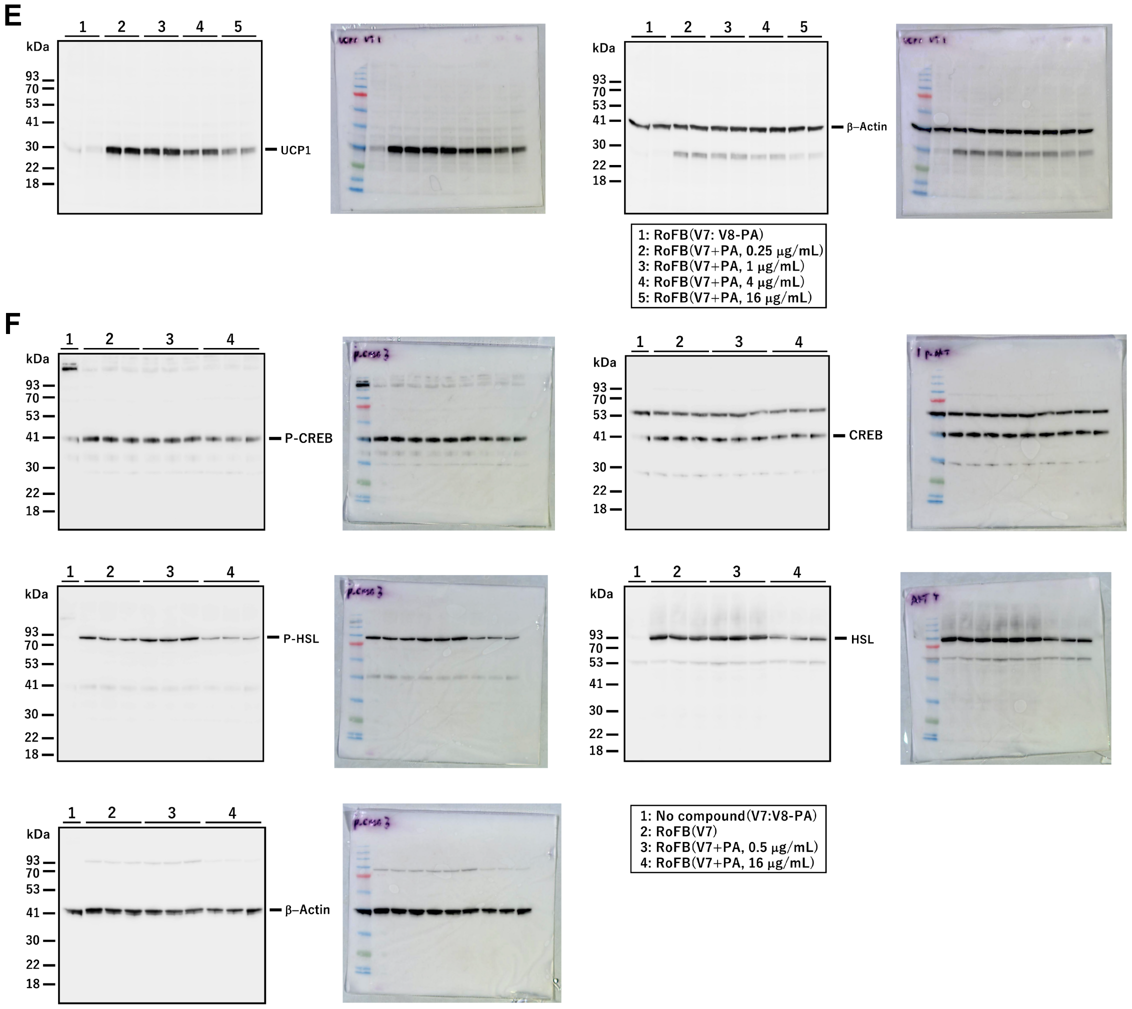

Supplementary Figure S8. Chemiluminescence and brightfield combined images of full-length western blots are shown in Figure 1B (**A**), Figure 1D (**B**), Figure 1F (**C**), Figure 2B (**D**), Figure 3C (**E**), and Figure 3H (**F**).

Supplementary Table S1. Sequences of primers used for qRT-PCR.

Gene	Sense primer	Antisense primer
TBP	ACTACGGGGTTATCACCTGTGAG	GTGCAGGAGTAGGCCACATTAC
UCP1	TCTACGACACGGTCCAGGAG	GAATACTGCCACTCCTCCAGTC
FABP4	GCCAGGAATTTGACGAAGTCA	CCCATTTCTGCACATGTACCAG
CIDEA	AAGGCCACCATGTATGAGATGTAC	ACAGGAACCGCAGCAGACTC
CKMT1	AGCAGGAATGGCTCGAGAC	ATCCTCCTCATTCACCCAGATC
CKMT2	TAACTGGCCGCAATGCTTC	ACCTCGGCACACACTTTCTG
CKB	GGCAACATGAAGGAGGTGTTC	CAGGTGAGGGTTCCACATG
ALPL	TGGACCTCGTTGACACCTG	GGGTCAAGGGTCAGGAGTTC
SLC6A8	GCAGCTACAACCGCTTCAAC	AGGATGGAGAAGACCACGAAG
